# Bioinformatic analysis of hippocampal histopathology in Alzheimer’s disease and the therapeutic effects of active components of traditional Chinese medicine

**DOI:** 10.3389/fphar.2024.1424803

**Published:** 2024-08-16

**Authors:** Chen Zhiyan, Zhan Min, Du Yida, He Chunying, Hu Xiaohua, Li Yutong, Wang Huan, Sun Linjuan

**Affiliations:** ^1^ Graduate School of Beijing University of Chinese Medicine, Beijing, China; ^2^ Department of Neurology, China Academy of Chinese Medical Sciences Xiyuan Hospital, Beijing, China; ^3^ China Academy of Chinese Medical Sciences, Beijing, China

**Keywords:** GEO dataset, bioinformatics, mitochondrial autophagy, echinacoside, SNCA, UBB

## Abstract

**Background and aim:**

Pathological changes in the central nervous system (CNS) begin before the clinical symptoms of Alzheimer’s Disease (AD) manifest, with the hippocampus being one of the first affected structures. Current treatments fail to alter AD progression. Traditional Chinese medicine (TCM) has shown potential in improving AD pathology through multi-target mechanisms. This study investigates pathological changes in AD hippocampal tissue and explores TCM active components that may alleviate these changes.

**Methods:**

GSE5281 and GSE173955 datasets were downloaded from GEO and normalized to identify differentially expressed genes (DEGs). Key functional modules and hub genes were analyzed using Cytoscape and R. Active TCM components were identified from literature and the Pharmacopoeia of the People’s Republic of China. Enrichment analyses were performed on target genes overlapping with DEGs.

**Result:**

From the datasets, 76 upregulated and 363 downregulated genes were identified. Hub genes included SLAMF, CD34, ELN (upregulated) and ATP5F1B, VDAC1, VDAC2, HSPA8, ATP5F1C, PDHA1, UBB, SNCA, YWHAZ, PGK1 (downregulated). Literature review identified 33 active components from 23 herbal medicines. Target gene enrichment and analysis were performed for six components: dihydroartemisinin, berberine, naringenin, calycosin, echinacoside, and icariside II.

**Conclusion:**

Mitochondrial to synaptic vesicle dysfunction pathways were enriched in downregulated genes. Despite downregulation, UBB and SNCA proteins accumulate in AD brains. TCM studies suggest curcumin and echinacoside may improve hippocampal pathology and cognitive impairment in AD. Further investigation into their mechanisms is needed.

## 1 Introduction

Alzheimer’s disease (AD) is a progressive neurodegenerative disorder and the most common form of dementia (Power et al., 2019). Over the past 3 decades, both the prevalence and mortality rates of AD have increased significantly (Li et al., 2022). In 2019, an estimated 55 million people worldwide were living with dementia, and the annual cost of diagnosing and treating AD reached $1.3 trillion (World Health Organization, 2023). This makes AD a leading cause of disability among older adults. The diagnosis of AD primarily relies on biomarkers such as amyloid-beta (Aβ) deposition, Tau pathology, and neurodegenerative or neuronal lesions (Jack et al., 2018), along with clinical manifestations including cognitive decline and psychoneurobehavioral changes (McKhann et al., 2011). When the cerebral cortex regions responsible for language and social behavior are affected, patients often lose the ability to live independently (National Institute on Aging). Retrospective studies have confirmed that AD lesions can be detected in the central nervous system (CNS) more than 20 years before the onset of clinical symptoms (Bateman et al., 2012), a stage defined as “preclinical AD” (Sperling et al., 2011). Following this prolonged period of preclinical decline, patients with AD eventually develop complex cognitive deficits (Monsell et al., 2014).

The hippocampus, as the brain structure where lesions are first detected, has received significant attention in the treatment of AD due to its prolonged pathological process. The hippocampus is crucial for learning and memory (Squire, 1992) and is involved in complex behavioral processes such as reinforcement learning and assisted decision-making (Ballard et al., 2019). Hippocampal lesions are considered a vital pathological process in AD. The accumulation of neurofibrillary tangles (NFTs) and Aβ plaques in the hippocampus is central to AD pathology (Calvo-Flores Guzmán et al., 2020). In the early stages of AD, damage to the connectivity between memory-related neurons in the hippocampus occurs (National Institute on Aging), and the degree of volumetric hippocampal atrophy is positively correlated with cognitive decline in AD patients (Mormino et al., 2009). Clinical studies have examined the microstructure of the hippocampus and assessed AD lesions using various analytical methods such as diffusion tensor imaging (DTI) (Zarei et al., 2013), seed-based correlation analysis (SCA) (Fellgiebel and Yakushev, 2011), and multivariate morphometric statistics (MMS) (Zheng et al., 2023). Clinical treatments for AD, including cholinesterase inhibitors and N-methyl-D-aspartate antagonists, alleviate clinical cognitive and behavioral symptoms but do not alter the progression of the disease (Passeri et al., 2022).

Traditional Chinese medicine (TCM) has a long history in treating dementia. Clinical studies and *in vitro* experiments have confirmed that many TCM therapies can alleviate cognitive symptoms in AD patients and improve the pathological damage associated with AD (Pei et al., 2020). TCM exerts a synergistic effect in AD patients by targeting multiple pathways involved in the pathological progression of AD (Zhang Y. et al., 2015), including the ubiquitin-proteasome pathway, the autophagy-lysosome pathway, and the NF-κB pathway (Ding et al., 2022).TCM has been proven effective in the early prevention of AD and in enhancing brain activity in individuals with AD (Zhang et al., 2019).

In this study, we focused on hippocampal tissue-specific expressed genes and comprehensively applied bioinformatics analyses to screen and identify hub genes and signaling pathways associated with AD hippocampal lesions. Additionally, we conducted a literature review to investigate the potential targets and mechanisms of action of TCM components in improving cognition in AD.

## 2 Materials and methods

### 2.1 Differentially expressed gene analysis

#### 2.1.1 Dataset retrieval process

**FIGURE 1:**
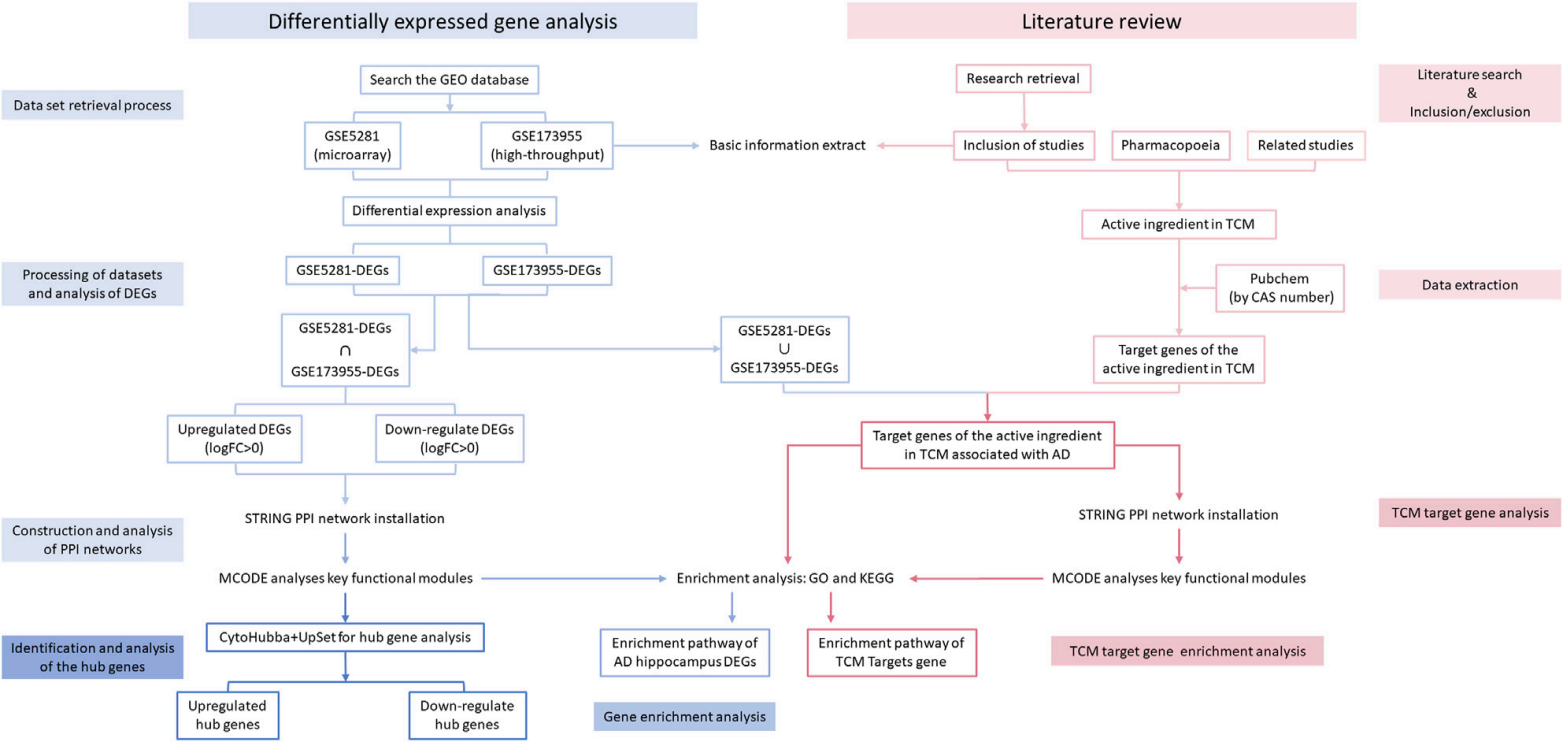
Flow diagram.

Figure 1 shows a flow diagram that outlines the various stages of our research process. In the Gene Expression Omnibus (GEO) database, the search terms “Alzheimer’s disease [MeSH Terms],” “Alzheimer’s disease,” “AD Alzheimer’s disease,” “AD,” “Alzheimer Dementia,” “Senile Dementia,” and “*Homo sapiens*” were used. The search aimed to obtain GSE (Gene Expression Omnibus Series) datasets, including high-throughput sequencing and microarray data, which were then screened according to the following criteria:

Inclusion criteria: 1) GSE dataset information or references specify inclusion criteria for AD and control samples; 2) GSE contains hippocampal tissue; 3) GSE includes both AD and control samples; 4) GSE data are RNA expression levels.

Exclusion criteria: No raw data available.

For sample selection of included GSE studies: 1) GSM (Gene Expression Omnibus Sample) samples of hippocampal tissue were included, and the total sample size should be greater than 10; 2) GSMs without clinical information were excluded.

#### 2.1.2 Processing of datasets and analysis of differentially expressed genes

Data pre-processing for the microarray dataset was performed using R-based software (R version 4.3.2). After reading the raw data with the “affy” package (Gautier et al., 2004), background correction was applied using the Robust Multiarray Average (RMA) method (Irizarry et al., 2003), and gene expression averages were calculated for the same gene probe detection results. Differential expression analysis was conducted using the “limma” package (Ritchie et al., 2015).

For high-throughput data, pre-processing was carried out using FastQC and Trimmomatic (Bolger et al., 2014) for quality control and assessment of raw data. After filtering out low-quality results, the data were converted to count form using Hierarchical Indexing for Spliced Alignment of Transcripts 2 (HISAT2) (Kim et al., 2015). Differential expression analysis was then performed using the “DESeq2” package (Love et al., 2014) (R version 4.3.2), and genes with an adjusted *p*-value <0.05 were selected as differentially expressed genes (DEGs). Heatmaps and volcano plots for each included dataset were generated using the “ggplot2” package (Wickham, 2016).

Changes in gene expression were assessed based on the sign of the log fold change (logFC). The intersection of upregulated and downregulated DEGs from each dataset was selected for further analysis.

#### 2.1.3 Gene enrichment analysis

Gene Ontology (GO) and Kyoto Encyclopedia of Genes and Genomes (KEGG) pathway analyses were performed using the ‘clusterProfiler’ package (Yu et al., 2012) (R version 4.3.2). Thresholds of *P* < 0.05 and q < 0.05 were set for both GO and KEGG analyses to identify pathways with significant differences. The results were visualized using the “enrichplot” (Yu, 2021) and “ggplot2” packages.

#### 2.1.4 Construction and analysis of protein-protein interaction networks

The intersection of DEGs from each GSE dataset was analyzed using the STRING (https://string-db.org/) online database (Szklarczyk et al., 2023) to construct protein-protein interaction (PPI) networks with moderate confidence (scores >0.4). The PPI networks were visualized using Cytoscape software (Shannon et al., 2003) (version 3.10.1). Cytoscape’s plug-in, Molecular Complex Detection (MCODE)(Bader and Hogue, 2003), was used to analyze key functional modules with the following selection criteria: K-core = 2, degree cutoff = 2, max depth = 100, and node score cutoff = 0.2. The “clusterProfiler” package was used for KEGG and GO analyses of the genes in each module.

#### 2.1.5 Identification and analysis of the hub genes

The cytoHubba plugin of Cytoscape (v 3.9.0) was used to score each node gene using 10 randomly selected algorithms, including Degree, EPC (Edge Percolated Component), MNC (Maximum Neighbourhood Component), BNC (Biological Network Centrality), BottleNeck, EcCentricity, Closeness, Betweenness, Clustering Coefficient, MCC (Maximum Clique Centrality), Radiality, and Stress (Chin et al., 2014). The top 15 hub genes from each algorithm were used to identify hub genes, which were then visualized using the ‘UpSetR’ package (Conway et al., 2017).

## 3 Literature review

### 3.1 Literature search

Studies were identified through a comprehensive search in the following databases: China National Knowledge Infrastructure (CNKI), Wan Fang database, SinoMed, China Science and Technology Journal Database, PubMed, Cochrane Library, and Web of Science. The search covered studies on TCM for AD published from the inception of the databases to January 2024, using a combination of subject terms and free terms. The results were included in EndNote X9 (version 3.3) for screening.

### 3.2 Inclusion/exclusion criteria

We reviewed experimental animal studies on the treatment of AD with TCM in rats and mice. The literature inclusion criteria were as follows: 1) The Morris water maze test showed a significant difference in behavioral tests between the treatment group and the model group (*p* < 0.05); 2) Hippocampal tissue analysis from the experimental animals showed a significant difference between the TCM treatment group and the model group (*P* < 0.05); 3) The therapeutic drug was a TCM active component or a single Chinese medicine decoction piece.

Exclusion criteria were as follows: 1) Animal models other than rats and mice; 2) AD models constructed by the investigators themselves through surgery.

For literature that does not list the specific chemical composition of TCM: 1) By comparing with the relevant parts of the Pharmacopoeia of the People’s Republic of China 2020 edition (Pharmacopoeia) (https://ydz.chp.org.cn/#/main), if the extraction method in the literature is the same as that in the Pharmacopoeia, the active components of TCM specified in the Pharmacopoeia were used for the analyses; 2) Relevant literature on the active components of the corresponding TCM was searched for assessment.

### 3.3 Data extraction

An initial screening was performed by two authors **(Du Yida, He Chunying)** based on the titles and abstracts of the literature retrieved from the database to exclude irrelevant studies, including clinical trials, reviews, non-TCM treatments, and pharmacological studies. The literature was then screened a second time using the full-text content based on the inclusion and exclusion criteria. Disagreements in the assessment of the content were adjudicated by a third author **(Zhan Min).**

Information on the design of the AD *versus* control model, sample size, therapeutic agents, and treatment duration of the included studies was extracted. Using *P* < 0.05 as a criterion, behavioral test results with significant differences and outcome indicators related to the hippocampus of experimental animals were screened as baseline information.

### 3.4 TCM target gene analysis

The Chemical Abstracts Service (CAS) (https://commonchemistry.cas.org/) or the National Drug Reference Standards (http://aoc.nifdc.org.cn/sell/home/) were used to search for the names of the TCM components in the included studies to obtain the CAS numbers. Both the CAS numbers and the names of the TCM components were searched in the PubChem database (https://pubchem.ncbi.nlm.nih.gov/) to identify the target genes. The target genes of the active components of TCM that were present in each DEG (adjusted *P*-value <0.05) of the included GSE datasets were selected. Core modules were analyzed for GO and KEGG enrichment using STRING to construct the PPI network, and Cytoscape’s MCODE plugin was used for further analysis. The target genes of the remaining TCM agents were directly enriched and analyzed as before.

## 4 Results

### 4.1 GSE dataset information and analysis of DEGs

A total of 35 patients from two GSE datasets (GSE5281, GSE173955) were included. Detailed information on test platforms, sample size, and patient age for these datasets is shown in the table below (Table 1).

**TABLE 1:** Detailed information on the GEO dataset.

GEO	Platform	Samples		Age	
		AD	Control	AD	Control
GSE5281 (Miller et al., 2008)	GPL570	10	13	77.8 ± 5.71	79.62 ± 9.4
GSE173955 (Carlsen and Hoffman, 2021)	GPL18460	6	9	91.75 ± 6.50	77 ± 8.96

### 4.2 Analysis of DEGs

**FIGURE 2:**
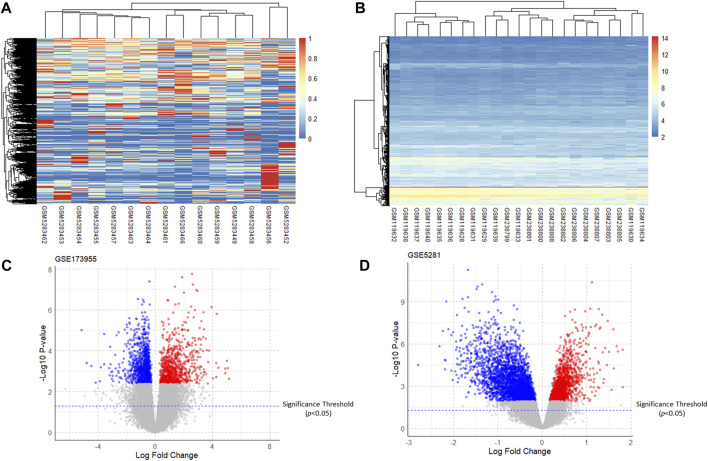
Heatmaps and volcano plots of the GSE dataset. **(A,B)**: Heatmaps of gene expression of GSE173955 and GSE5281; **(C,D)**: Volcano plots of DEGs of GSE173955 and GSE5281 (adjusted *P*-value < 0.05).

After analyzing GSE5281 using the R software “limma” package and GSE173955 using the “DESeq2” package, the DEGs of the two GSE datasets were obtained. The heatmap and volcano plot of the two GSE datasets are shown in (Figure 2). Taking the intersection of DEGs from the two GSE datasets resulted in 76 upregulated genes (adjusted *P*-value <0.05, logFC >0) and 363 downregulated genes (adjusted *P* -value <0.05, logFC <0). For the logFC value and the adjusted *P* -value of upregulated and downregulated genes, see (Supplementary Material S2).

### 4.3 Results of enrichment analysis

GO annotations of DEGs consisted of three categories: CC (Cellular Component), BP (Biological Process), and MF (Molecular Function), which were used to analyze DEG functional enrichment. KEGG analysis was conducted to determine the relationship between DEGs and signaling pathways.

After GO and KEGG enrichment analyses of all upregulated gene processes, only the results of the biological process and molecular function categories of the GO analysis were obtained. The upregulated genes are involved in epidermal cell differentiation and developmental pathways in terms of biological processes, while in terms of molecular function, they are associated with prostaglandin signaling (PTGIR, PTGER1) (Table 2).

**TABLE 2:** Results of partial enrichment analysis of upregulated genes.

Enrichment analysis	GO term	Function	Genes	FDR adjusted *P*-value
GO_BP	GO:0045606*	positive regulation of epidermal cell differentiation	NUMA1/FOXC1/BMP4/ALOX15B	3.38E−3
GO_MF	GO:0004955*	prostaglandin receptor activity	PTGIR/PTGER1	0.04

FDR: false discovery rate.

^*^Enrichment results have adjusted *P*-values <0.01.

After cluster analysis of the downregulated genes by MCODE, the higher-scoring clustering modules were subjected to GO and KEGG enrichment analyses. The top 10 significantly enriched terms in each GO category were identified (Benjamini and Yosef, 1995).

**FIGURE 3:**
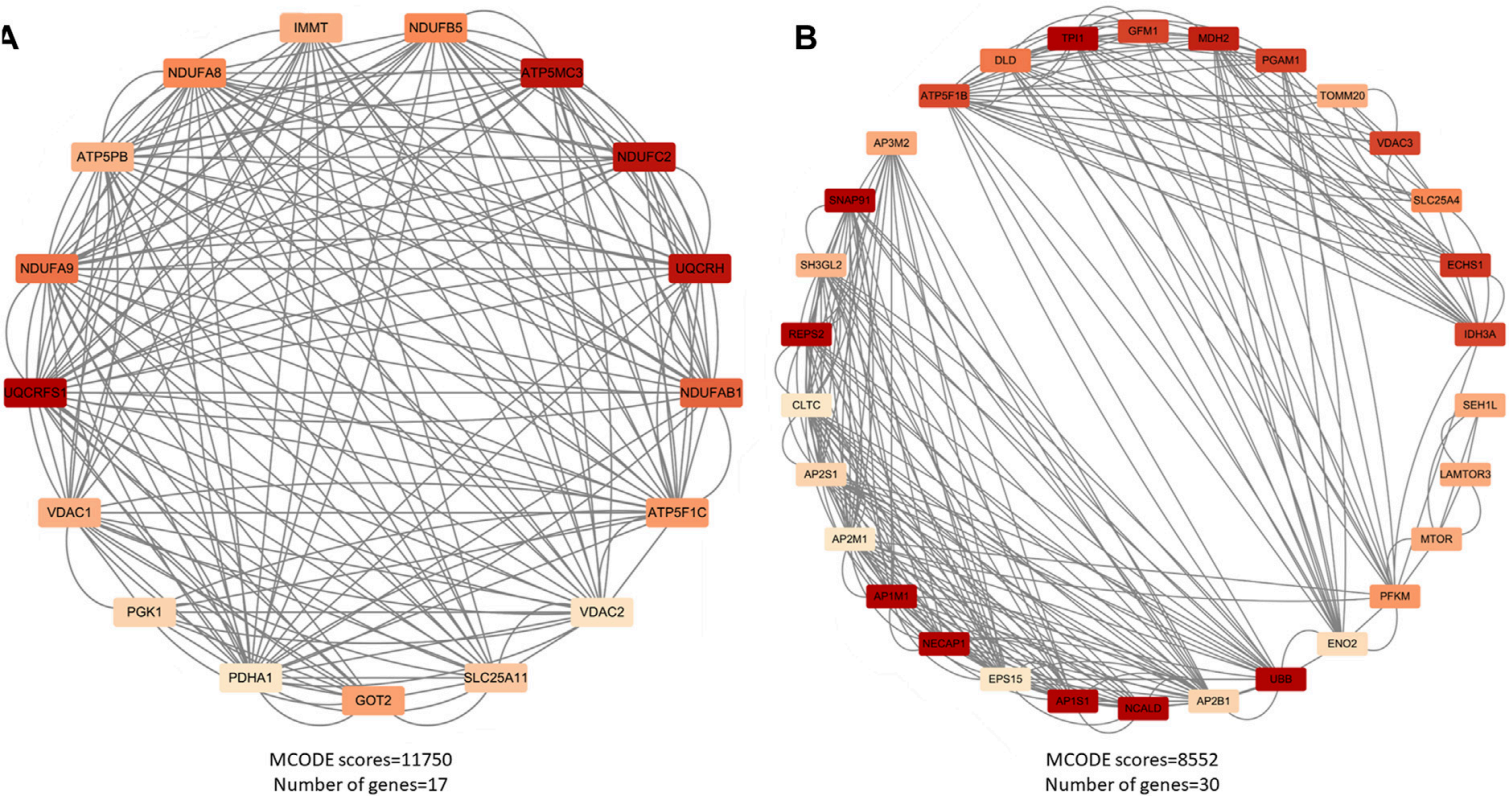
Major functional modules of downregulated genes. **(A)** Downregulated gene module 1 (MCODE score = 11750, number of genes = 17); **(B)** Downregulated gene module 2 (MCODE score = 8552, number of genes = 30).

The enrichment results were broadly classified into three categories: 1) Mitochondrial structure-function related pathways, including mitochondrial ribosomes, mitochondrial gene expression, and mitochondrial endomembrane composition; 2) Cellular energy metabolism pathways, including cellular respiration, ATPase activity, and oxidative phosphorylation; 3) Synaptic vesicle related pathways. See (Table 3) and (Figure 3). This is consistent with previous studies that found mitochondrial damage and presynaptic vesicle loss in the synapses of nerve cells in AD patients, and the two often influence each other (Wang et al., 2023). In addition, downregulated genes were enriched for a variety of degenerative neuropathies, including Alzheimer’s disease, Parkinson’s disease, prion diseases, and Huntington’s disease. For specific results of enrichment analyses of upregulated genes and individual modules of downregulated genes, see (Supplementary Material S3).

**TABLE 3:** Enrichment analysis of partial clustering modules of downregulated genes.

MCODE scores and number of genes	Enrichment analysis	GO/KEGG term	Function	Genes
Scores = 11750 Genes = 17	GO_BP*	GO:0009060	aerobic respiration	PDHA1/ATP5F1C/NDUFAB1/ATP5PB/UQCRH/NDUFA8/NDUFC2/NDUFB5/NDUFA9/UQCRFS1
		GO:0015986	proton motive force-driven ATP synthesis	ATP5F1C/NDUFAB1/ATP5PB/ATP5MC3/NDUFA8/NDUFC2/NDUFB5/NDUFA9
	GO_CC*	GO:0098798	mitochondrial protein-containing complex	PDHA1/VDAC1/IMMT/ATP5F1C/NDUFAB1/ATP5PB/ATP5MC3/UQCRH/NDUFA8/NDUFC2/NDUFB5/NDUFA9/UQCRFS1
	GO_MF*	GO:0015453	oxidoreduction-driven active transmembrane transporter activity	UQCRH/NDUFA8/NDUFC2/NDUFB5/NDUFA9/UQCRFS1
	KEGG*	hsa05012	Parkinson disease	VDAC1/ATP5F1C/NDUFAB1/ATP5PB/VDAC2/ATP5MC3/UQCRH/NDUFA8/NDUFC2/NDUFB5/NDUFA9/UQCRFS1
		hsa05020	Prion disease	
		hsa05016	Huntington disease	
		hsa05010	Alzheimer disease	
Scores = 8552 Genes = 30	GO_BP*	GO:0048488	synaptic vesicle endocytosis	AP2B1/AP2M1/AP2S1/AP3M2/SH3GL2/SNAP91
	GO_CC*	GO:0030118	clathrin coat	AP1M1/AP1S1/AP2B1/AP2M1/AP2S1/AP3M2/CLTC/EPS15/NCALD/NECAP1
	GO_MF*	GO:0030276	clathrin binding	AP1M1/AP1S1/AP2B1/AP2M1/AP2S1/CLTC/NCALD/SNAP91
	KEGG*	hsa01200	Carbon metabolism	DLD/ECHS1/ENO2/IDH3A/MDH2/PFKM/PGAM1/TPI1
Scores = 6000 Genes = 6	GO_BP*	GO:0032543	mitochondrial translation	MRPS23/MRPL44/MRPS33/MRPS35/MRPL47/MRPS17
	GO_CC*	GO:0005761	mitochondrial ribosome	
	GO_MF*	GO:0003735	structural constituent of ribosome	MRPS23/MRPS33/MRPS35/MRPL47/MRPS17
Scores = 6000 Genes = 6	GO_BP*	GO:0097401	synaptic vesicle lumen acidification	ATP6V1E1/ATP6V1C1/ATP6AP1/ATP6V0D1/ATP6V1B2/ATP6V1A
		GO:0016188	synaptic vesicle maturation	
	GO_CC*	GO:0033176	proton-transporting V-type ATPase complex	
	GO_MF*	GO:0042625	ATPase-coupled ion transmembrane transporter activity	ATP6V1E1/ATP6V1C1/ATP6V0D1/ATP6V1B2/ATP6V1A

^*^Enrichment results have adjusted *P*-values <0.01.

### 4.4 PPI network analysis and identification of hub genes

**FIGURE 4:**
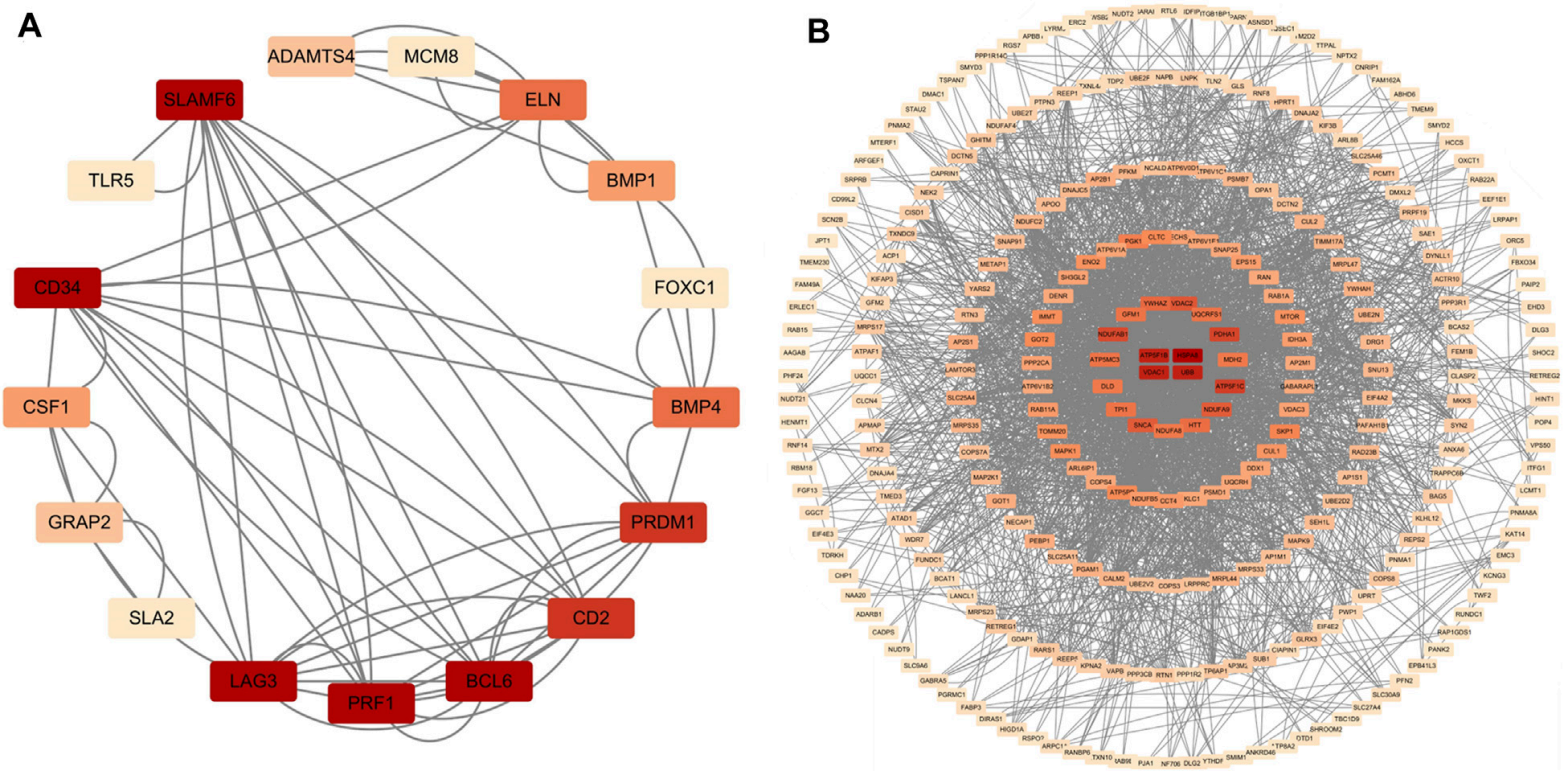
PPI network diagram of up- and downregulated genes. **(A)** Upregulated genes; **(B)** Uownregulated genes.

**FIGURE 5:**
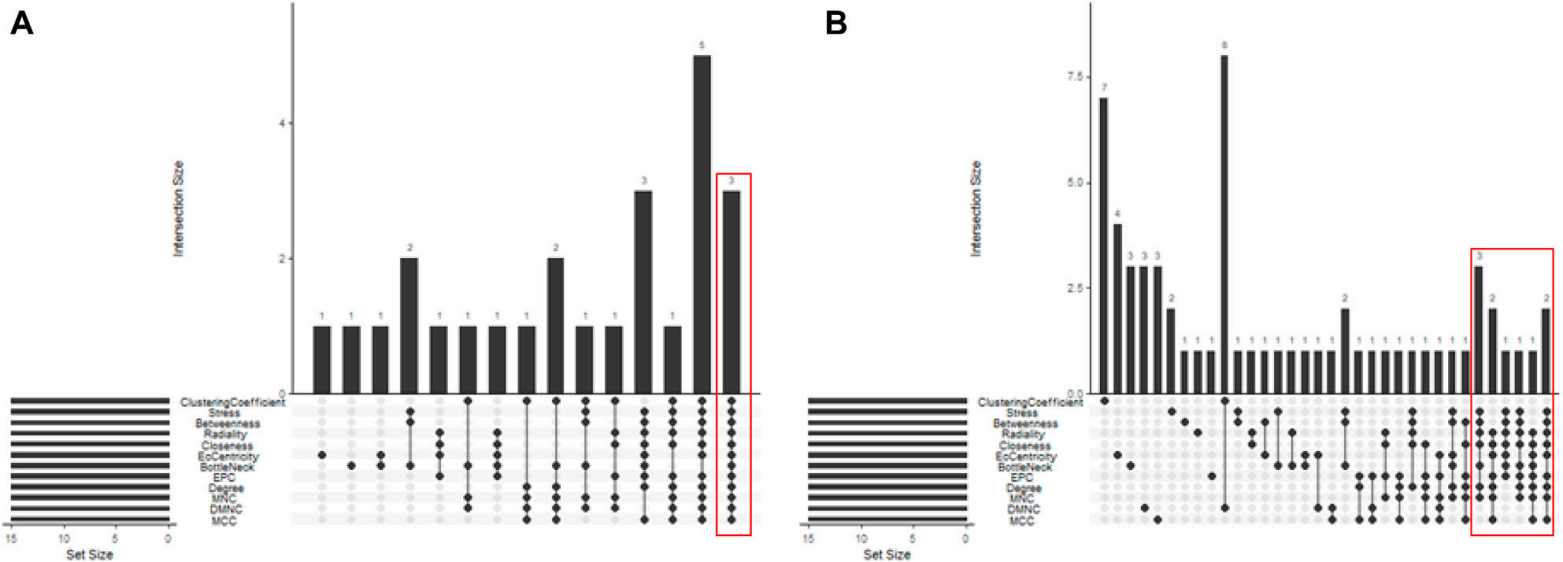
Screening of up- and downregulated hub genes using the “UpSetR” package. The highlighted regions in red boxes indicate the hubgenes identified through the “UpSetR” package. **(A)** Upregulated genes; **(B)** Downregulated genes.

In total, 76 upregulated genes and 363 downregulated genes were analyzed separately to characterize potential PPIs using the online STRING database. PPIs with a moderate confidence score ≥0.4 were selected and then imported into Cytoscape for further complex network analysis (Figure 4). The MCC, DMNC, MNC, Degree, EPC, BottleNeck, EcCentricity, Closeness, Radiality, Betweenness, Stress, and Clustering Coefficient algorithms of CytoHubba were used to integrate and rank the hub genes. The top 15 hub genes from each algorithm were used to screen hub genes through the ‘UpSetR’ package (Figure 5).

**FIGURE 6:**
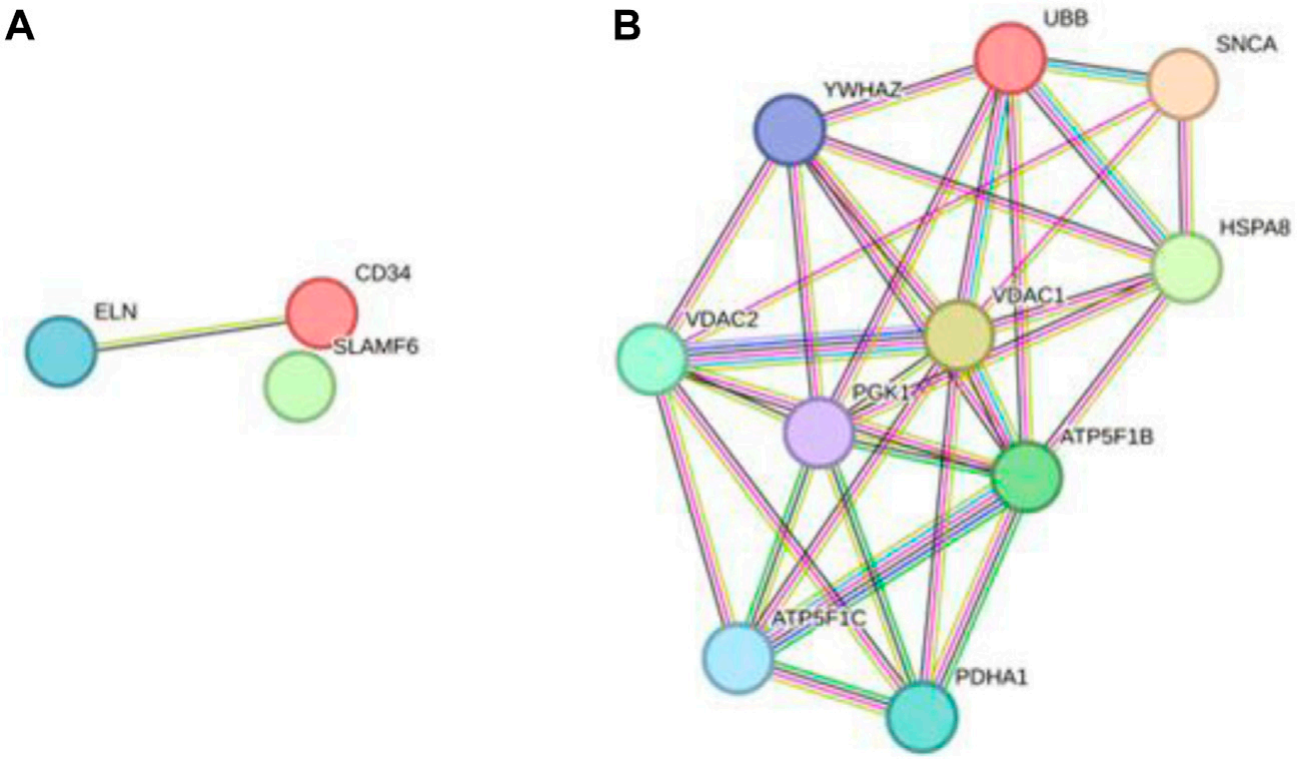
PPI network of hub genes. **(A)** upregulated genes; **(B)** downregulated genes.

Ultimately, three upregulated hub genes (SLAMF, CD34, ELN) and ten downregulated hub genes (ATP5F1B, VDAC1, VDAC2, HSPA8, ATP5F1C, PDHA1, UBB, SNCA, YWHAZ, PGK1) were filtered, uploaded to STRING, and mapped (Figure 6). After determining the hub genes, we used the UniProt Knowledgebase/Swiss-Prot (UniProtKB/Swiss-Prot) database to retrieve detailed information and provide comments about these genes, as shown in (Table 4).

**TABLE 4:** Both up- and downregulated proteins.

Gene symbol	UniProtKB/Swiss-Prot	Protein name	GSE5281		GSE173955	
			LogFC	Adjusted *P*-value	LogFC	Adjusted *P*-value
SLAMF6	Q96DU3	SLAM family member 6	0.79	0.00	1.51	0.04
CD34	P28906	Hematopoietic progenitor cell antigen CD34	0.56	0.00	1.36	0.02
ELN	P15502	Elastin	0.38	0.01	1.19	0.02
ATP5F1B	P06576	ATP synthase subunit beta, mitochondrial	−1.92	0.00	−0.60	0.01
VDAC1	P21796	Voltage-dependent anion-selective channel protein 1	−0.73	0.01	−0.77	0.00
VDAC2	P45880	Voltage-dependent anion-selective channel protein 2	−1.22	0.01	−0.45	0.04
HSPA8	P11142	Heat shock cognate 71 kDa protein	−1.73	0.00	−0.70	0.02
ATP5F1C	P36542	ATP synthase subunit gamma, mitochondrial	−1.45	0.00	−0.42	0.05
PDHA1	P08559	Pyruvate dehydrogenase E1 component subunit alpha, somatic form, mitochondrial	−1.12	0.00	−0.45	0.05
UBB	P0CG47	Polyubiquitin-B	−0.63	0.01	−0.32	0.03
SNCA	P37840	Alpha-synuclein	−0.85	0.02	−1.07	0.02
YWHAZ	P63104	14-3–3 protein zeta/delta	−1.21	0.00	−0.83	0.02
PGK1	P00558	Phosphoglycerate kinase 1	−1.03	0.00	−0.74	0.00

### 4.5 Included studies

**FIGURE 7:**
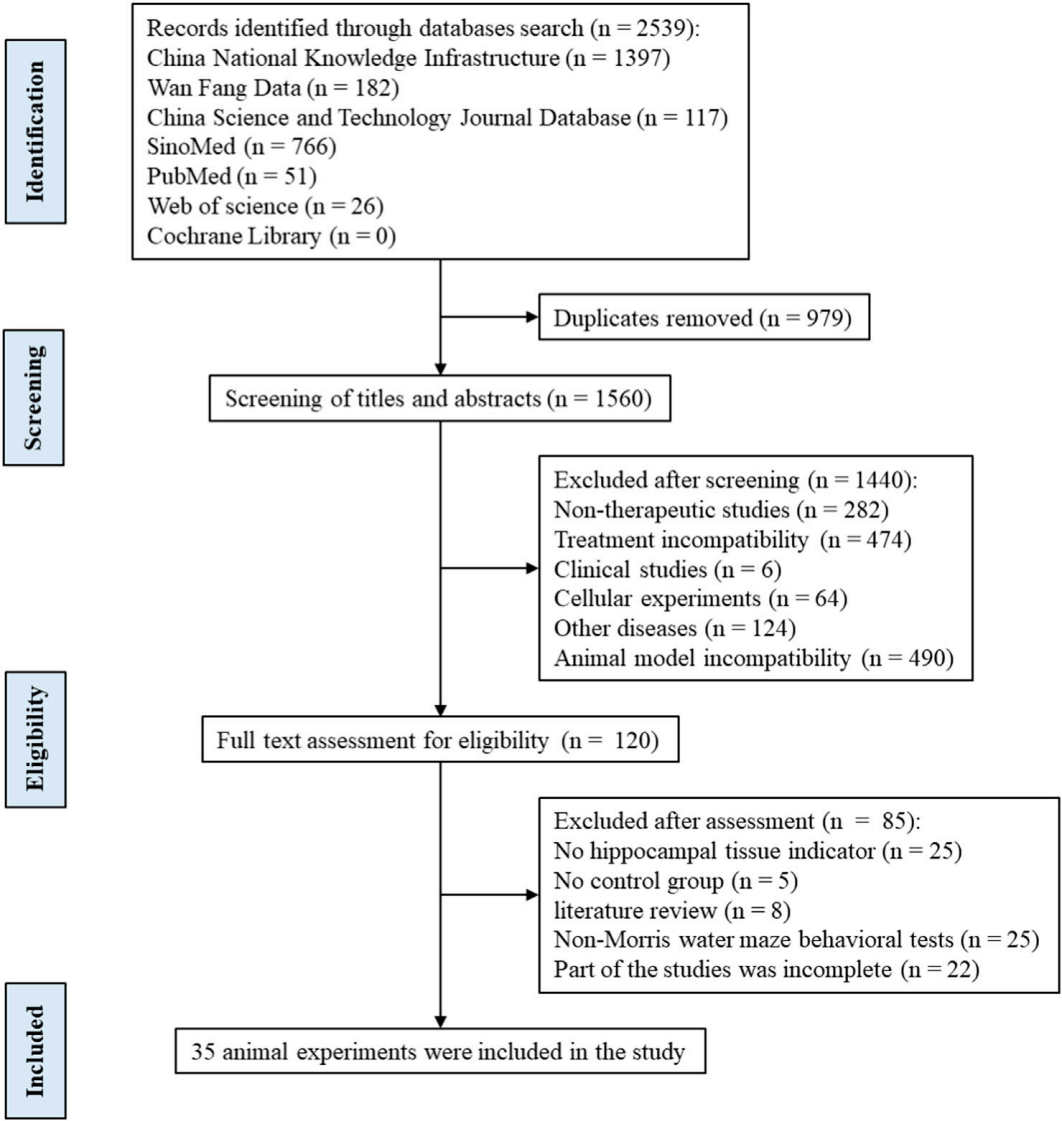
Flow diagram of the search and selection of studies.

Following the differential expression analysis of hippocampal tissue samples from AD patients, we explored the effects of TCM on hippocampal pathology in AD animal models. After rigorously and systematically searching the databases, 1560 unique records were identified in EndNote, and the titles and abstracts were screened. The full texts of 120 articles were assessed for eligibility, and 35 studies were included in this study (Figure 7). See (Supplementary Material S4) for the baseline information from the included studies. The risk of bias in the included studies was assessed using the 10 items of SYRCLE’s Risk of Bias Tool (Hooijmans et al., 2014), with the results presented in (Supplementary Material S5).

**FIGURE 8:**
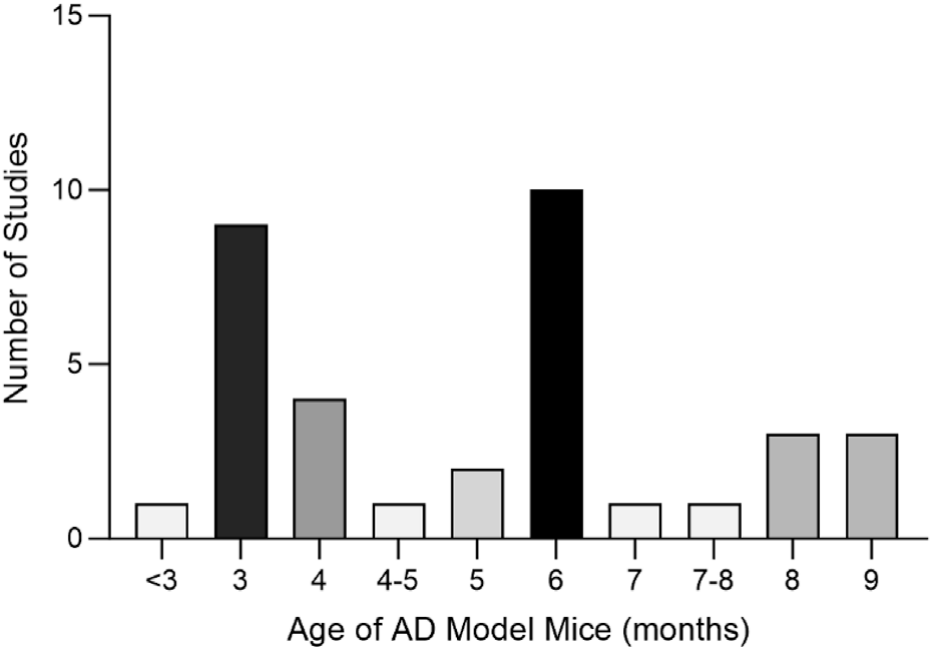
Age Distribution of Experimental Animals in the Included Studies Altogether, nine active components of TCM were not found as target genes in PubChem: β-asarone (Nie, 2011), tetrahydroxystilbene glucoside (Chen C., 2017), schisandrin (Piao et al., 2019), smilagenin (Yang, 2019), safflower yellow B (Shi et al., 2018), 3,6′-disinapoyl sucrose (Wang XF. et al., 2021), pinoresinol diglucoside (Yu, 2018), rehmannioside D (Zhao, 2022), and onjisaponin B (Li et al., 2016). Valganciclovir (Liu, 2019) and neoeriocitrin (Gu, 2018), on the other hand, lack gene targets from humans.

A total of 35 papers were included (Zhou, 2009; Wang H., 2011; Wang P. W., 2011; Nie, 2011; Wei, 2013; Zhang Z.-W. et al., 2015; Dang, 2015; Fan, 2015; Li, 2015; Li et al., 2016; Yuan, 2016; Chen C., 2017; Chen Y. W., 2017; Gao, 2017; Yan, 2017; Yang, 2017; Gu, 2018; Liu, 2018; Shi et al., 2018; Yu, 2018; Chen, 2019; Liu, 2019; Piao et al., 2019; Yang, 2019; Zhang, 2019; Zhu, 2019; Kong F. G., 2020; Kong Y. Y., 2020; Jiang et al., 2020; Ranran et al., 2020; Wang XF. et al., 2021; Wu, 2021; Wang, 2022; Zhao, 2022; He, 2023). Of these, 32 papers used APP/PS1 mice as AD model animals and C57/BL6J mice or wild-type mice as controls. Three papers used Senescence-Accelerated Mouse Prone 8 (SAMP8) mice as AD model animals and Senescence-Accelerated Mouse Resistant 1 (SAMR1) mice as controls. All animals were 3–9 months old. Among them, 10 (28.57%) were less than 3 months old, 17 (48.57%) were 3–6 months old, and 6–9 (22.86%) were 6–9 months old, as shown in (Figure 8). In total, 33 active components from 23 TCMs were included. Thirteen active components from seven TCMs were identified by reviewing Pharmacopoeia and other relevant studies. See (Supplementary Material S6) for the Latin names of TCMs and the active components of TCMs in English and Chinese.

### 4.6 Efficacy of active components of TCM

Analyzing the changes in hippocampal tissue-related indices from the included studies revealed that various active components of TCM effectively improved Aβ deposition and Tau phosphorylation lesions in hippocampal tissue. These TCM components ameliorate histopathological damage in the hippocampus of AD model mice through various pathways, such as regulating glial cell activity, attenuating oxidative stress, reducing cellular inflammation, and acting on the insulin and estrogen pathways.

Based on the results of the enrichment analyses of DEGs, various TCM components were identified with mechanisms of action related to mitochondrial autophagy and synaptic function.

For late-stage AD model mice, TCM treatments improved neuronal autophagy and synaptic function. Geniposide (6 months old) (Zhang, 2019) enhanced autophagy by inhibiting the mTOR signaling pathway, improving hippocampal lesions in AD model animals, as evidenced by increased expression of LC3-II and Beclin1, and decreased p62 protein levels. Total alkaloids of *Dendrobium nobile* Lindl. (7 months old) (Yang, 2017) and tetrahydroxy-stilbene glucoside (4–5 months old) (Chen C., 2017) increased synaptophysin protein content in hippocampal tissue, improving synaptic function. Additionally, tetrahydroxy-stilbene glucoside has a protective effect on nerve cells and is effective in neurological diseases such as Parkinson’s disease and vascular dementia (Wang et al., 2019).

Various TCM components demonstrated therapeutic effects at the neuronal and hippocampal tissue levels in AD model mice at different pathological stages: 1) For neuronal inflammation, drugs such as calycosin (7–8 months old) (Gao, 2017) and curcumin (8 months old) (Chen Y. W., 2017) reduced inflammatory responses in hippocampal neuronal cells; 2) In hippocampal tissue, components such as dihydroartemisinin (6 months old) (He, 2023), berberine (6 months old) (Wu, 2021), total alkaloids of *D. nobile* Lindl. (7 months old) (Yang, 2017), and forsythoside B (8 months old) (Kong F. G., 2020) modulated glial cell activity; curcumin (3 months old) (Wang H., 2011; Dang, 2015), tetrahydroxy-stilbene glucoside (4–5 months old) (Chen C., 2017), and total alkaloids of *D. nobile* Lindl. (7 months old) (Yang, 2017) improved synaptic function in hippocampal neuronal cells; 3) Some TCM components are related to hormonal pathways: curcumin (3 months old) (Wang H., 2011; Dang, 2015) and valganciclovir (6 months old) (Liu, 2019) modulated the insulin pathway, while extracts of *Eucommia ulmoides* Oliv. (3 months old) (Yu, 2018), *Drynaria roosii* Nakaike (3 months old) (Gu, 2018), and *Cullen corylifolium* (L.) Medik. (3 months old) (Liu, 2018) exerted neuroprotective effects through the estrogenic pathway; 4) Extracts of *Rehmannia glutinosa* (Gaertn.) DC (4 months old)(Zhao, 2022) and dihydroartemisinin (6 months old) (He, 2023) repaired blood-brain barrier damage.

### 4.7 Analysis of the target genes of the active components of TCM

A total of 30.79% of the target genes of the included components were found in the DEGs of the GSE173955 and GSE5281 datasets, with the exception of geniposide (Zhang, 2019), forsythoside A (Wang, 2022), and notopterol (Jiang et al., 2020) (Table 5). Among them, dihydroartemisinin (He, 2023) had the highest percentage (49.53%) and number (53) of target genes in DEGs. The downregulated hub genes are included in the target genes of dihydroartemisinin (He, 2023) (HSPA8, PGK1) and echinacoside (Yuan, 2016) (SNCA). The TCM components with a high number of target genes contained in the DEGs were analyzed using the MCODE plug-in, and then the clustering module was used for GO and KEGG pathway enrichment. We searched the UniProt Knowledgebase/Swiss-Prot database to annotate the target genes of each active component of TCM. See (Supplementary Material S7).

**TABLE 5:** Target genes included in the DEGs of GSE173955 and GSE5281 of active components of TCM.

Active components of TCM	Target genes		Target genes included in DEGs (GSE173955 and GSE5281)	
	Total	DEGs	Upregulated genes	Downregulated genes
Dihydroartemisinin	107	53	CSRP1/MYC/RELA/AIM2/HP1BP3/KHSRP/NFKBIA/SF1/ZYX	HSPA8/MAPK1/MDH1/PGK1/TPI1/ACTG1/ALDH7A1/ATP5F1A/ATP5MG/ATP5PO/CCT3/DDIT3/DPYSL2/EEF1A1/EGR1/ENO1/GAPDH/GPI/HNRNPA2B1/HNRNPK/KEAP1/LDHA/LDHB/LGALS1/NPM1/PFN1/PPIA/PRDX1/RPL10/RPL14/RPL18/RPL23A/RPL35/RPL4/RPS17/RPS19/RPS9/SFPQ/SNRPD2/SOD1/SOD2/SRSF4/TPM1/TUBA1A
Berberine	189	48	AURKC/CDK2/CEBPB/FAS/H2BC8/H3C10/ITGA1/RELA/SETDB1/SIRT1/BIRC5/EGFR/EIF4EBP1/EP300/H3C7/H4C3/IGF1R/KDM4A/KDM6B/LINC00943/NCF1/NFKBIA/SETD1B/SETD3/SIRT2/TOP1	BNIP3/BNIP3L/CYCS/CYP1A1/FUNDC1/IDH3A/MAPK8/SMYD3/UBE2B/CTNNB1/CTSD/CTSL/DZIP3/HAT1/HGF/KDM1A/KDM5B/MAP1LC3B/NCF2/NSD2/PARP1/PRMT6
Naringin	58	17	BMP4/CDK2/FAS/RELA/VCAM1/CCL5/CYBA/CYP1B1/EGFR	CYCS/CYP1A1/MAPK1/CTNNB1/HRAS/MT3/SLCO1B3/TSLP
Calycosin	44	15		MAPK1/PEBP1/PGAM1/PRDX2/SKP1/BLVRB/CDK7/CDKN2D/ERP29/NME1/PNPO/POLB/PRDX1/RBX1/UGT1A8
Verbascoside	49	13	ARNT/CDK2/RELA/SNAI1/VCAM1/EGFR/KDM4A/POLK/RB1	CYP1A1/MAPK1/SMAD2/SOD2
Icariside II	32	9	EGFR/SLC10A1	GOT1/MAPK1/MTOR/PDPK1/BRAF/NOS2/PARP1
Psoralen	25	6	NFKB1	MAPK8/MTOR/ATF4/DDIT3/HSD17B10
Echinacoside	12	5	NTRK1/BACH1	MAPK1/SNCA/DDIT3
Icariin	19	4	RELA/SIRT1	SLCO1B3/SOD2
Hydroxyl safflower yellow A	11	4	RELA/VCAM1/NFKBIA	HK1
1-deoxynojirimycin	10	4	GANC	GAA/GANAB/MAN1B1
Curcumin	18	3		ABCC5/PTGS1/TUBA3D
(−)-Dendrobine	11	3		GPX1/KEAP1/SOD2
paeoniflorin	10	3	VCAM1	BNIP3/PTGS1
Imperatorin	9	3	CYP1B1/PTPN7	DUSP3
Catalpol	9	1	SIRT1	
Isoimperatorin	9	1	CYP1B1	
Forsythoside B	4	1	BACH1	
Isopsoralen	4	1	NFKB1	

## 5 Dihydroartemisinin (DHA)

In animal experiments (He, 2023), DHA, the first-generation derivative of artemisinin, was found to inhibit the TLR4/MyD88/NF-kB pathway, reduce the inflammatory response in the hippocampal tissue of 6-month-old AD model mice, and decrease the permeability of the blood-brain barrier. The visualization results of the target genes of DHA are shown in (Table 6). The enrichment analysis results and the upregulation and downregulation of target genes in the differential expression analysis mentioned earlier are presented in (Table 7).

**TABLE 6:** Network diagram of dihydroartemisinin gene target gene PPI and enrichment results.

Network diagram of PPI	Results of MCODE analysis		
	Clustering module	Genes	Network diagram of clustering module
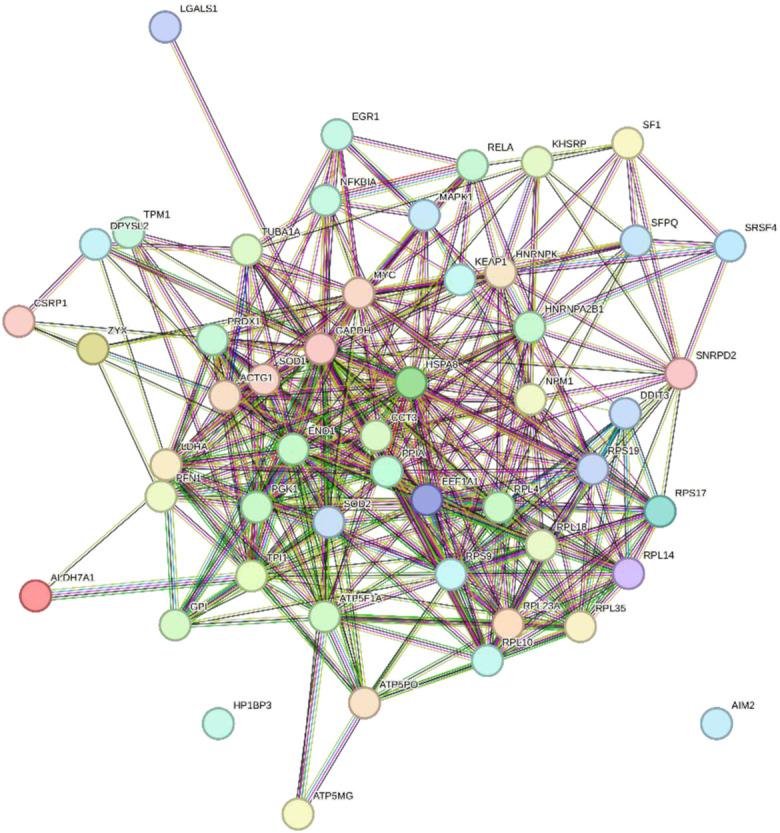	Scores = 10778 Gene numbers = 19	DDIT3/ENO1/HNRNPA2B1/HSPA8/LDHA/PFN1/PGK1/PRDX1/RPL10/RPL14/RPL18/RPL23A/RPL35/RPL4/RPS17/RPS19/RPS9/SOD1/TPI1	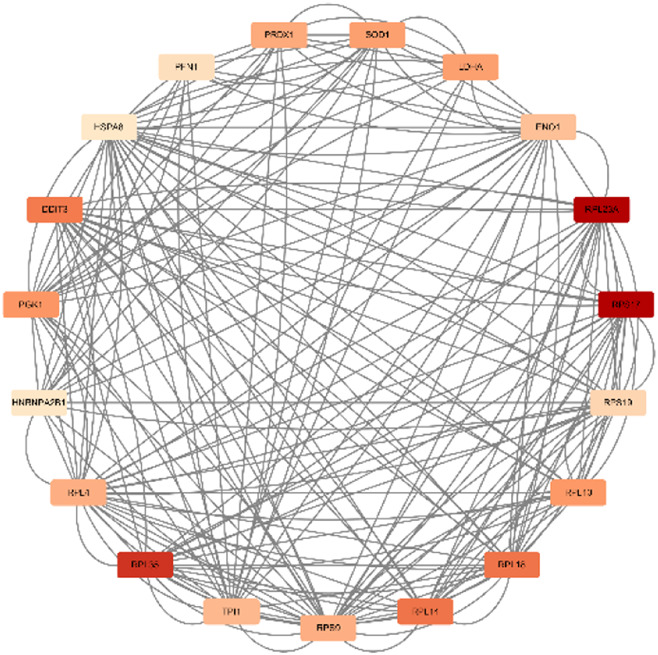
	Score = 7636 Gene numbers = 12	ATP5F1A/NPM1/CCT3/MYC/GPI/ACTG1/EEF1A1/GAPDH/SOD2/PPIA/HNRNPK/TUBA1A	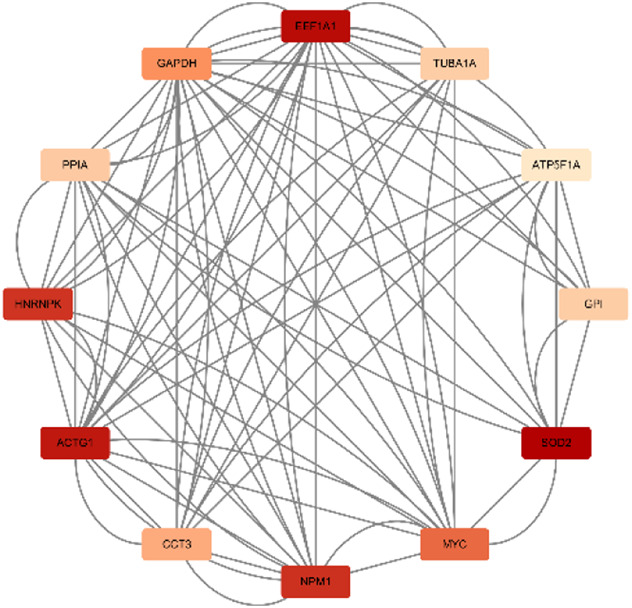

**TABLE 7:** Results of enrichment analysis of dihydroartemisinin.

MCODE analysis	Enrichment analysis	GO/KEGG term	Function	Genes	
				Up	Down
Scores = 10778 Gene number = 19	GO_BP	GO:0042254*	ribosome biogenesis		RPL10/RPL14/RPL23A/RPL35/RPS17/RPS19/RPS9
	GO_CC	GO:0022626*	cytosolic ribosome		RPL10/RPL14/RPL18/RPL23A/RPL35/RPL4/RPS17/RPS19/sRPS9
	GO_MF	GO:0003735*	structural constituent of ribosome		RPL10/RPL14/RPL18/RPL23A/RPL35/RPL4/RPS17/RPS19/RPS9
	KEGG	hsa03010*	Ribosome		RPL10/RPL14/RPL18/RPL23A/RPL35/RPL4/RPS17/RPS19/RPS9
		hsa04066*	HIF-1 signaling pathway		ENO1/LDHA/PGK1
Scores = 7636 Gene number = 12	GO_BP	GO:2001242*	regulation of intrinsic apoptotic signaling pathway	MYC	HNRNPK/PPIA/SOD2

^*^Enrichment results have adjusted *P*-values <0.01.

The target genes of DHA are enriched in pathways related to ribosomal structure and the Hypoxia-inducible factor 1 (HIF-1) signaling pathway and are downregulated in the DEGs. According to PubChem’s target gene information, cell experiments have confirmed that DHA interacts with various ribosomal proteins. Additionally, DHA interacts with Enolase 1 (ENO1), L-lactate dehydrogenase A (LDHA), and Phosphoglycerate Kinase 1 (PGK1), which are involved in the HIF-1 signaling pathway (Ravindra et al., 2015), though the mechanisms of action require further investigation. Furthermore, DHA targets HNRNPK and PPIA (Ravindra et al., 2015), promoting the degradation of Myc protein (Wang et al., 2015), which is associated with the endogenous apoptosis signaling pathway. DHA also promotes ROS generation in Molt-4 cells, thereby inducing an increase in SOD2, reflecting its cytotoxic effects (Bhatt et al., 2021).

The earliest mention of *Artemisia annua* L. for the treatment of various types of malaria was in the Zhouhou Beiji Fang. Currently, DHA is widely used in the clinical treatment of malaria and plays a powerful role in inhibiting a variety of inflammation-related diseases (Yu et al., 2021). Chinese medicine considers *A. annua* L. to have a cooling effect, commonly used in the treatment of febrile diseases (Zhang et al., 2016). This study found that MYC is an upregulated DEG in AD, consistent with the co-localization of phosphorylated c-Myc with abnormal Tau protein deposition in AD pathology (Ferrer et al., 2001a). DHA selectively induced the degradation of c-Myc in a proteasome-dependent manner in tumor cells overexpressing c-Myc (Lu et al., 2010), but its effect on c-Myc in neuronal cells still requires further investigation. Additionally, DHA can alleviate lipopolysaccharide-induced neuroinflammation and reduce neuronal damage in the hippocampus of mice by inhibiting the PI3K/AKT signaling pathway (Gao et al., 2020).

Additionally, DHA has been shown to reduce blood-brain barrier permeability in a sepsis model by increasing the expression of the tight junction protein occludin (OCLN) (Liu et al., 2022). However, the mechanisms by which it affects blood-brain barrier permeability in AD still require further investigation.

## 6 Berberine

Berberine, an active component in the TCM Coptis, has been shown to inhibit the endoplasmic reticulum stress PERK/eIF2α pathway (Wu, 2021), reducing Aβ42 deposition and Tau hyperphosphorylation in the hippocampal neurons of 6-month-old APP/PS1 mice. The visualization results of the target genes of berberine are shown in (Table 8), and the enrichment analysis results are presented in (Table 9).

**TABLE 8:** Network diagram of berberine gene target gene PPI and enrichment results.

Network diagram of PPI	Results of MCODE analysis		
	Clustering module	Genes	Network diagram of clustering module
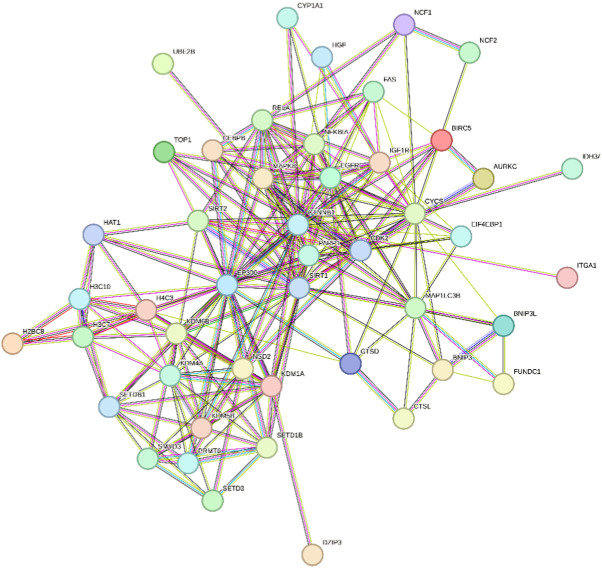	Scores = 10000 Gene numbers = 12	MAPK8/CTNNB1/RELA/EP300/CDK2/EGFR/MAP1LC3B/CEBPB/FAS/SIRT1/NFKBIA/PARP1	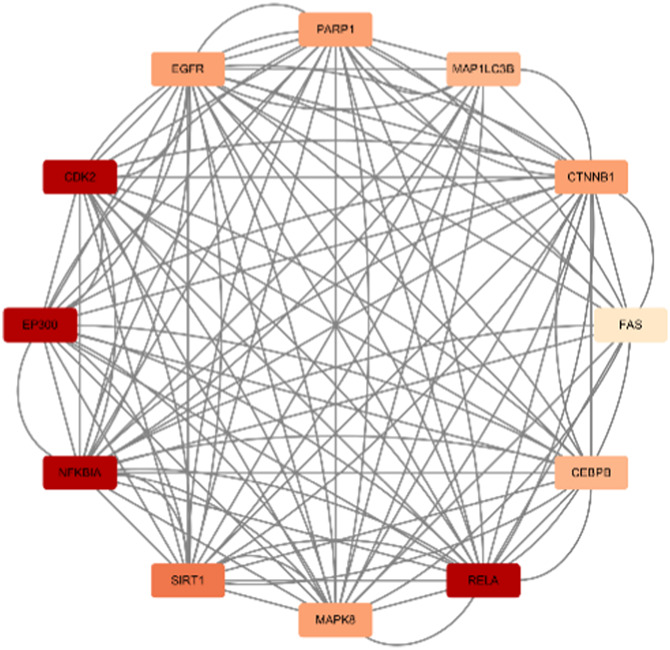
	Score = 4286 Gene numbers = 8	HAT1/H3C7/H3C10/KDM4A/KDM6B/PRMT6/SMYD3/SETD1B	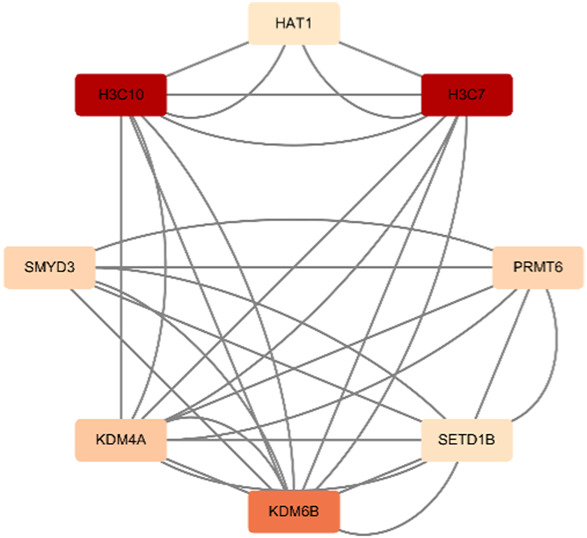

**TABLE 9:** Results of enrichment analysis of berberine.

MCODE analysis	Enrichment analysis	GO/KEGG term	Function	Genes	
				Up	Down
Scores = 10000 Gene number = 12	GO_BP	GO:0009411*	response to UV	RELA/EGFR/SIRT1/EP300	MAPK8/PARP1
	GO_CC	GO:0062197*	cellular response to chemical stress	RELA/EGFR/FAS/SIRT1	MAPK8/PARP1
	GO_MF	GO:0009416*	response to light stimulus	RELA/EGFR/SIRT1/EP300	MAPK8/PARP1
Scores = 4286 Gene number = 8	KEGG	hsa04613*	Neutrophil extracellular trap formation	H3C7/H3C10	HAT1

^*^Enrichment results have adjusted *p*-values <0.01.

According to the target gene information from PubChem, in a Parkinson’s disease cell model, berberine inhibits the activation of the NF-κB pathway by modulating the LINC00943/miR-142-5p/KPNA4 axis, thereby reducing the nuclear translocation of the transcription factor p65 (RELA) (Li et al., 2021). Additionally, other target genes are linked to berberine’s mechanisms in treating various cancers. Specifically, genes enriched in the neutrophil extracellular trap (NET) pathway, such as H3C7, H3C10, and HAT1, play a role in berberine’s therapeutic effects on acute myeloid leukemia (Wang et al., 2016).

Berberine exerts neuroprotective effects through various molecular mechanisms by modulating multiple signaling pathways, including NF-κB and JNK (Tian et al., 2023). Research on berberine and its target genes reveals that in a lipopolysaccharide-stimulated RAW264.7 macrophage model, berberine inhibits the NF-κB signaling pathway by activating SIRT1 (Zhang et al., 2017) and exerts anti-inflammatory effects via the p300 (EP300)/p65lys310 axis (Zhang S. et al., 2023). Additionally, EGFR exhibits significant immunoreactivity around neuroplaque in AD patients (Birecree et al., 1988). Molecular docking analysis shows stable binding between berberine and the EGFR target, although no significant differences in EGFR mRNA levels were observed in the brain tissues of AD model mice (3 × Tg AD mice) (Wei et al., 2023).

Modern Chinese medicine believes that the pathogenesis of AD can be summarized as “toxins damaging brain collaterals” (Su et al., 2011). The 2023“Guideline for the Diagnosis and Treatment of Alzheimer’s Disease with Integrated Chinese and Western Medicine” suggests that Huanglian Jiedu Decoction (with Coptis as the principal Chinese medicine decoction piece) can clear heat and detoxify, improving cognition in patients with MMSE scores ≤10. Other studies have found that berberine reduces Aβ production by promoting autophagy (Huang et al., 2017), decreases abnormal Tau protein phosphorylation (Chen Y. et al., 2020), and alleviates mitochondrial dysfunction, thereby improving AD pathology (Wong et al., 2021).

## 7 Naringin

The included studies found that *D. roosii* Nakaike extract with more than 50% flavonoid content exerted neuroprotective effects through the ERβ/P38 pathway (Gu, 2018), reducing cellular inflammation and neuronal apoptosis in the hippocampus, and increasing levels of choline acetyltransferase (ChAT) and acetylcholine (Ach). Referring to the Pharmacopoeia, we consider naringenin as the active component in *D. roosii* Nakaike.

Naringin is enriched in pathways related to the cellular response to tumor necrosis factor, protein kinase activator activity, membrane raft, and lipid and atherosclerosis (Table 10). Among the enriched genes, CYP1B1 and CYBA are associated with ROS production in AD pathology. CYP1B1, as part of the cytochrome P450 family, promotes oxidative stress in AD (Chen YY. et al., 2020), and cell experiments have confirmed that naringin is a weak inhibitor of CYP1B1 (Shimada et al., 2010). CYBA is an important component of the NADPH oxidase complex (Afsar et al., 2023), responsible for ROS generation, and naringin can inhibit the TNF-α-induced overexpression of CYBA mRNA and protein (Li et al., 2014). The target genes in other enriched pathways are derived from studies on naringin’s mechanisms in treating various cancers.

**TABLE 10:** Results of enrichment analysis of naringin.

Enrichment analysis	GO/KEGG term	Function	Genes	
			Up	Down
GO_BP	GO:0071356*	cellular response to tumor necrosis factor	FAS/RELA/VCAM1/CCL5/CYP1B1	MAPK1
GO_CC	GO:0045121*	membrane raft	FAS/EGFR	MAPK1/CTNNB1
GO_MF	GO:0030295*	protein kinase activator activity	CCL5/EGFR	MT3
KEGG	hsa05417*	Lipid and atherosclerosis	FAS/RELA/VCAM1/CCL5/CYBA	CYCS/CYP1A1/MAPK1/HRAS

^*^Enrichment results have adjusted *P*-values <0.01.

Multiple behavioral tests have confirmed that naringin, the active component in Drynaria roosii Nakaike, can improve cognition in AD model animals (Poudineh et al., 2022). Naringin has been shown to ameliorate AD neurocyte structural lesions through mechanisms such as reducing Aβ and Tau protein phosphorylation (Meng et al., 2021), modulating the glutamate system (Wang et al., 2013), and chelating excess metals (Prakash et al., 2013). Chinese medicine believes that *D. roosii* Nakaike has the effect of tonifying the kidneys and strengthening bones. Clinically, it is often used in TCM to treat skeletal diseases such as osteoporosis, osteoarthritis, and fractures (Deng et al., 2022), as well as oral bone-related diseases such as periodontitis and pulpitis (Huang et al., 2023). In an early case report, oral administration of *D. roosii* Nakaike alone improved limb weakness and cognitive impairment in an AD patient (Yang, 2004).

## 8 Calycosin

*In vivo*, calycosin (active component of *Astragalus propinquus* Schischkin) has antioxidative stress and anti-inflammatory neuroprotective effects on the hippocampal neurons of 7–8 month old APP/PS1 mice. *In vitro*, calycosin can attenuate Aβ-induced oxidative stress and inflammation through the PKC/Nrf2 pathway (Gao, 2017).

The active component of *Astragalus propinquus* Schischkin, calycosin, targets genes in hepatocellular carcinoma cells that are enriched in pathways related to mitigating intracellular oxidative stress (Zhang et al., 2013) (Table 11). Other studies have shown that intraperitoneal injection of calycosin in APP/PS1 mice reduces hippocampal Aβ, Tau, interleukin-1beta (IL-1β), tumor necrosis factor-alpha (TNF-α), acetylcholinesterase, and malondialdehyde levels in a dose-dependent manner (Song et al., 2017). TCM posits that AD involves a deficiency of qi and blood (Li and Gao, 2017), and *Astragalus propinquus* Schischkin is commonly used in TCM clinical practice for treating AD due to its qi-tonifying and yang-raising properties according to Pharmacopoeia. *Astragalus propinquus* Schischkin is frequently prescribed in TCM for AD treatment (Song et al., 2019). Other active components of Astragalus, primarily including astragaloside IV (Wu Y. et al., 2021) and astragalus polysaccharides (Yao et al., 2014), have shown potential in ameliorating AD pathology by inhibiting Aβ production and aggregation, preventing neuronal damage or apoptosis, and reducing neuroinflammation (Wang et al., 2024).

**TABLE 11:** Results of enrichment analysis of calycosin

Enrichment analysis	GO/KEGG term	Function	Genes	
			Up	Down
GO_BP	GO:0019430*	removal of superoxide radicals		PRDX2/PRDX1
GO_MF	GO:0004601*	peroxidase activity		PRDX2/PRDX1
KEGG	hsa00260*	Glycine, serine and threonine metabolism		PGAM1

^*^Enrichment results have adjusted *p*-values <0.01.

## 9 Verbascoside and echinacoside

According to the Pharmacopoeia, both verbascoside and echinacoside are recognized as active components of cistanche (*Cistanche deserticola* Y.C.Ma or *Cistanche tubulosa* (Schenk) Wight). Prophylactic administration of cistanche glycosides increased the resistance of hippocampal neuronal cells to oxidative damage in 5-month-old AD model mice (Yuan, 2016). We performed GO and KEGG enrichment analyses to further investigate the potential roles of the target genes of verbascoside and echinacoside.

Verbascoside targets genes are enriched in pathways related to cellular oxidative stress and aging (Table 12). It inhibits the overexpression of SOD2 mRNA induced by oxidized LDL (Kostyuk et al., 2011). Target genes such as ARNT, MAPK1, RELA (Potapovich et al., 2011), and EGFR (Pastore et al., 2012) are associated with skin inflammatory responses. Other target genes have been identified through cell experiments in prostate cancer (Wu CH. et al., 2021) and acute promyelocytic leukemia (Lee et al., 2007).

**TABLE 12:** Results of enrichment analysis of verbascoside.

Enrichment analysis	GO/KEGG term	Function	Genes	
			Up	Down
GO_BP	GO:0034599*	cellular response to oxidative stress	ARNT/RELA/EGFR	MAPK1/SOD2
GO_CC	GO:0005721#	pericentric heterochromatin	SNAI1/KDM4A	
GO_MF	GO:0031625*	ubiquitin protein ligase binding	RELA/EGFR/KDM4A/RB1	SMAD2
KEGG	hsa04218*	Cellular senescence	CDK2/RELA/RB1	MAPK1/SMAD2

^*^Enrichment results have adjusted *p*-values <0.01.

^#^Enrichment results have adjusted *p*-values <0.05.

Echinacoside is enriched in pathways related to neurological disorders, including synaptic transmission and the regulation of neuronal apoptosis (Table 13). Echinacoside significantly increases the phosphorylation levels of TrkA (NTRK1) and ERK2 (MAPK1) (Zhu et al., 2013), activating the extracellular signal-regulated kinase (ERK) signaling pathway to protect neurons. Additionally, it modulates the ROS/ATF3/CHOP pathway to reduce the expression of apoptotic genes DDIT3 (CHOP) and SNCA (α-synuclein, α-syn), thereby inhibiting apoptosis in Parkinson’s disease cell models (Zhao et al., 2016).

**TABLE 13:** Results of enrichment analysis of echinacoside.

Enrichment analysis	GO/KEGG term	Function	Genes	
			Up	Down
GO_BP	GO:0050806*	positive regulation of synaptic transmission	NTRK1	MAPK1/SNCA
	GO:0043523*	regulation of neuron apoptotic process	NTRK1	SNCA/DDIT3
GO_CC	GO:0005770*	late endosome	NTRK1	MAPK1/DDIT3
KEGG	hsa04210*	Apoptosis	NTRK1	MAPK1/DDIT3
	hsa05010*	Alzheimer disease		MAPK1/SNCA/DDIT3

^*^Enrichment results have adjusted *p*-values <0.01.

Upon reviewing the literature, we found that the precursor of nerve growth factor (pro-NGF) is increased in the hippocampus (Hock et al., 2000), while the expression of NGF and the TrkA gene (NTRK1) is decreased in cholinergic basal forebrain neurons (Mufson et al., 2008; Mufson et al., 1995), potentially related to reduced retrograde transport of NGF (Schindowski et al., 2008). Additionally, studies have shown that the expression levels of ERK2 (MAPK1) in the hippocampus of AD patients remain unchanged, but phosphorylated ERK2 is increased (Ferrer et al., 2001b; Khezri et al., 2023), which is associated with Tau protein phosphorylation (Kerr et al., 2006). Lewy-related pathology (LRP) in the brains of AD patients is immunohistochemically co-localized with α-syn (SNCA) and Tau pathology (Twohig and Nielsen, 2019a). While α-syn is involved in multiple presynaptic mechanisms, endogenous α-syn depletion has been found to exacerbate neurodegeneration (Chandra et al., 2005).

Our differential expression analysis identified DDIT3 as a downregulated DEG (adjusted *P*-value = 0.001234713 < 0.05) in GSE5281, which contradicts other studies showing increased expression of CHOP (DDIT3) and other ER stress-related proteins in the hippocampus of AD patients. This discrepancy may be related to the unfolded protein response (UPR) (Ismael et al., 2021): UPR upregulates CHOP via the PERK/eIF2α/ATF4 pathway (Hetz and Mollereau, 2014), with activation occurring in the early stages of neurofibrillary degeneration (Hoozemans et al., 2009), while the GSE5281 samples represent late-stage AD pathology (Braak stages V-VI) (Miller et al., 2008).

TCM posits that “the kidney stores essence and essence houses will,” with kidney deficiency being a primary pathogenesis of cognitive impairment in AD (Zhang M. et al., 2023). According to the Pharmacopoeia, *Cistanche deserticola* Y.C.Ma has properties of tonifying the kidneys and aphrodisiac, invigorating the blood, and moisturizing the intestines and acting as a laxative. It is included in multiple TCM formulations for treating AD (Wang X. et al., 2021; Dong, 2017; Qi et al., 2017; Ma F. Y., 2018) and can alleviate the common symptom of constipation in AD patients (Wang et al., 2022). Other animal studies have shown that total glycosides of Cistanche inhibit neuronal apoptosis and enhance free radical scavenging in AD model mice (Gu et al., 2016). Calycosin’s neuroprotective effects are associated with regulating neuroinflammation via the NF-κB-p65 pathway (Chen et al., 2022), while echinacoside modulates the PERK/eIF2α pathway to alleviate ER stress in APP/PS1 mice (Dai et al., 2020).

## 10 Icariside II

In 9-month-old APP/PS1 mice, icariin II inhibits BACE1 protein levels by regulating hippocampal and cortical PERK/eIF2α phosphorylation levels and PPARγ protein expression, thereby reducing Aβ production. It also inhibits PDE5A expression to protect neuronal cells (Yan, 2017). GO analysis of icariin II target genes revealed enrichment in pathways related to peptide hormone response, protein kinase activity, and cell-substrate adhesion. KEGG analysis indicated involvement in cancer-related pathways.

According to PubChem, Icariin II affects enriched target genes involved in the mechanisms of prostate cancer (Lee et al., 2009), non-small cell lung cancer (Song et al., 2012), and osteosarcoma (Geng et al., 2014) in disease model cells (Table 14). *In vivo* and *in vitro* studies have found that Icariin II reduces neuronal damage in AD model animals by activating the BDNF/TrkB/CREB signaling pathway (Liu et al., 2018) and alleviates LPS-induced neuroinflammation by inhibiting the TLR4/MyD88/NF-κB pathway (Zhou et al., 2019).

**TABLE 14:** Results of enrichment analysis of icariin II.

Enrichment analysis	GO/KEGG term	Function	Genes	
			Up	Down
GO_BP	GO:0043434*	response to peptide hormone		GOT1/MAPK1/MTOR/PDPK1/BRAF/PARP1
GO_CC	GO:0005925#	focal adhesion	EGFR	MAPK1/PDPK1
GO_MF	GO:0004674*	protein serine/threonine kinase activity	EGFR	MAPK1/MTOR/PDPK1/BRAF/

^*^Enrichment results have adjusted *p*-values <0.01.

Chinese medical theory posits that “the kidney nourishes marrow, and the brain is the sea of marrow.” As one ages, the essence and qi in the kidneys gradually decline, leading to brain marrow deficiency and resulting in cognitive impairments such as memory loss (Li et al., 2006). *Epimedium brevicornu* Maxim. is known for its function of “tonifying the kidney and essence” (Long et al., 2024) and is frequently mentioned in literature analyzing the usage patterns of Chinese medicine decoction pieces for treating AD (Wu S. et al., 2021; Sun et al., 2022; Zhong et al., 2021).

## 11 Other active components of TCM

Some of the target genes of the active components of TCM included in the study intersect minimally with DEGs but have effects in reducing Aβ deposition while inhibiting neuroinflammation in APP/PS1 mice. Among these, 1-Deoxynojirimycin has been shown to reduce the expression of neuroinflammatory factors in the hippocampal tissue of 3-month-old SAMP8 mice, possibly through the upregulation of the BDNF/TrkB signaling pathway in the hippocampus (Chen, 2019). Psoralen extract may alleviate the expression of inflammatory factors in the hippocampal tissue of 3-month-old APP/PS1 mice via the ERβ/ERK signaling pathway (Liu, 2018). Additionally, molecular docking studies have indicated that psoralen, an active components of *Psoralea corylifolia* L. (Somani et al., 2015), and derivatives of 1-Deoxynojirimycin possess cholinesterase inhibitory activity (Ahuja-Casarín et al., 2021).

Multiple studies included in the literature have investigated the mechanisms of curcumin in treating AD. Curcumin, an active component of the Chinese medicine decoction piece *Curcuma longa* L., has been reported to improve insulin signaling pathway disorders in 3-month-old APP/PS1 mice by activating the PI3K/AKT pathway downstream of insulin receptor substrate 1 (IRS-1), thereby reducing Aβ expression and the formation of Aβ oligomers (ADDLs) in the hippocampal CA1 region (Wang P. W., 2011; Dang, 2015). In 8-month-old APP/PS1 mice, curcumin treatment lowered the levels of IL-1β and TNF-α in the hippocampus and inhibited neuroinflammatory responses (Chen Y. W., 2017), suggesting that curcumin has therapeutic effects in both early and late stages of AD pathology.

Further studies revealed that curcumin can reduce Aβ-activated microglial inflammatory cytokine mRNA and protein levels by inhibiting the ERK1/2 and p38 kinase signaling pathways (Shi et al., 2015). Additionally, curcumin activates the Nrf2 pathway and inhibits the nuclear translocation of NF-κB, reducing LPS-induced NF-κB luciferase activity (Fagiani et al., 2020). Curcumin also binds to Aβ, increasing the helical propensity of Aβ peptides (Salamanova et al., 2021) and inhibiting Aβ aggregation, which suggests its potential as an early diagnostic probe for AD (Chen et al., 2018).

It is currently believed that glycogen synthase kinase-3β (GSK-3β) links senile plaques and neurofibrillary tangles in the pathological changes of AD. *In vitro* experiments have shown that Aβ activates GSK-3β signaling (Takashima et al., 1996a; Takashima et al., 1996b), leading to abnormal APP processing and synaptic dysfunction (Deng et al., 2014). Additionally, GSK-3β, as one of the Tau kinases (Fuster-Matanzo et al., 2012), directly associates with Tau protein to form functional units (Sun et al., 2002; Chun et al., 2004). Hyperphosphorylated Tau further activates GSK-3β by increasing oxidative stress, neuroinflammation, and apoptosis (Saeki et al., 2011).

Curcumin inhibits Aβ-induced activation of GSK-3β in human neuroblastoma SH-SY5Y cells. The specific mechanism involves curcumin reducing the mRNA and protein expression of PTEN, a negative regulator of PIP3, which in turn increases the levels of the PIP3 phosphorylated substrate Akt. Activated Akt, as an upstream regulator of GSK-3β, inhibits the phosphorylation of GSK-3β at the Ser9 site (Huang et al., 2014).

## 12 Discusion

This study selected GSE datasets (GSE5281, GSE173955) with clearly diagnosed AD, hippocampal tissue samples, and sample sizes of ≥10. We found that the DEGs in the hippocampal tissue of AD patients are enriched in pathways related to mitochondrial structure, mitochondrial energy metabolism, and synaptic vesicles. Using the Cytoscape plugin cytoHubba combined with the “UpSet” package, we identified hub genes, and identified the upregulated hub genes (SLAMF6, CD34, ELN) and downregulated hub genes (ATP5F1B, VDAC1, VDAC2, HSPA8, ATP5F1C, PDHA1, UBB, SNCA, YWHAZ, PGK1). Additionally, we reviewed studies that investigated the improvement of cognitive impairment and hippocampal tissue pathology in AD model animals after treatment with TCM. We analyzed the mechanisms by which these active components of TCM ameliorate AD pathology.

Among the upregulated hub genes, SLAMF6 belongs to the signaling lymphocytic activation molecule (SLAM) family receptors, which are expressed in various immune cells, including T cells, B cells, and NK cells (Yigit et al., 2019). It is a susceptibility gene for systemic lupus erythematosus (Zhong and Veillette, 2008). The upregulation of SLAMF6 may be related to the complex changes in immune cells during neurodegenerative diseases (Heneka, 2020).

The cell-surface protein CD34 is a marker for vascular endothelial cells, hematopoietic progenitor cells, and endothelial progenitor cells (Nielsen and McNagny, 2008). Studies have shown that the number of circulating CD34^+^ bone marrow progenitor cells (BMPCs) decreases with age (Hajjar et al., 2016). However, an increase in circulating CD34^+^ progenitor cells has been observed in the peripheral blood of early AD patients (Bigalke et al., 2011). Additionally, the counts of circulating CD34^+^ BMPCs and early endothelial progenitor cells (EPCs) are negatively correlated with MMSE scores in patients with moderate to severe AD (Stellos et al., 2010). Research suggests that the upregulation of CD34 may be associated with the repair of vascular injury in the central nervous system and treatment with cholinesterase inhibitors (Romaus-Sanjurjo et al., 2023).

The ELN gene encodes elastin, a component of the extracellular matrix (ECM) in the central nervous system, which contributes to the mechanical strength and elasticity of the ECM. Elastin plays a crucial role in neurogenesis, neuronal migration, and other neural cell structures and activities (Ma et al., 2020). As elastin ages, it degrades into elastin-derived peptides (EDPs). *In vivo* and *in vitro* experiments confirm that elastin-like peptides (ELPs) significantly increase Aβ levels in the hippocampus of AD model mice and AD model cells (Ma et al., 2019), suggesting that an increase in EDPs is associated with the pathological progression of AD. Furthermore, the degradation of elastin in the leptomeningeal arterioles of AD patients increases progressively from Braak stages II to VI, while degradation is less pronounced in medium-sized arteries (Merlini et al., 2016). It is hypothesized that the upregulation of ELN may help maintain the ECM and the structure of small blood vessels in the central nervous system.

Among the downregulated hub genes involved in cellular energy metabolism, phosphoglycerate kinase (PGK) is a central nervous system glycolytic enzyme. Its isoform, PGK1, acts as a rate-limiting enzyme in the second phase of glycolysis (Danshina et al., 2010), regulating energy production and redox balance. Pyruvate dehydrogenase E1 component subunit alpha (PDHA1) is a key component of the pyruvate dehydrogenase complex (PDC)(Patel et al., 2014; Patel et al., 2014), linking glycolysis to the tricarboxylic acid cycle in mitochondria. The downregulation of PGK1 and PDHA1 aligns with the metabolic dysfunction observed in AD pathology. Current studies indicate that significantly reduced glucose metabolism in brain regions such as the hippocampus and temporal cortex is a precursor symptom of AD (Mosconi et al., 2008), involving impaired glycolytic function (An et al., 2018). Furthermore, decreased pyruvate dehydrogenase complex activity has been found in the prefrontal cortex of AD patients, which is negatively correlated with the clinical dementia rating (CDR) (Bubber et al., 2005). The role of PGK1 in AD pathology requires further investigation. Some studies suggest that activation of PGK1 promotes autophagic degradation of various pathological aggregates (Chen et al., 2023), PGK1 can bind to Aβ *in vitro* and co-deposit in plaques (Rahman et al., 2015; Drummond et al., 2017).

In terms of mitochondrial energy metabolism, the downregulated hub genes ATP5F1B and ATP5F1C encode subunits of ATP synthase (Jonckheere et al., 2012), which are crucial components of mitochondrial ATP synthesis. The downregulation of ATP5F1B and ATP5F1C is associated with mitochondrial dysfunction in AD patients. Numerous studies have shown extensive mitochondrial abnormalities in the AD brain (Swerdlow, 2018), including reduced expression of ATP synthase in the hippocampal tissue of AD patients (Schägger and Ohm, 1995). Another study found a 61% reduction in the expression of mitochondrial electron transport chain subunit genes in the hippocampal CA1 region compared to controls (Liang et al., 2008). Regarding mitochondrial structure, the voltage-dependent anion channel (VDAC) is a pore-forming protein on the outer mitochondrial membrane and a key participant in mitochondria-mediated apoptosis (Shoshan-Barmatz et al., 2010). VDAC is also a primary regulator of metabolite exchange between the cytosol and mitochondria, with its isoforms including VDAC1 and VDAC2 (Zinghirino et al., 2021). In transgenic mice, interactions between VDAC1 and Aβ and phosphorylated Tau were observed in the cortex and hippocampus (Manczak and Reddy, 2012), which may contribute to mitochondrial dysfunction in AD pathogenesis. VDAC also co-localizes with full-length APP and Aβ in the frontal cortex of AD patients, and VDAC1 levels progressively increase in the cortex (Manczak and Reddy, 2012). While many studies suggest that reduced expression of VDAC1 may benefit synaptic activity and improve AD pathology (Manczak et al., 2013), it has been found that VDAC1 expression varies across different brain regions in AD patients (Yoo et al., 2001). The mechanisms by which VDAC affects AD and its potential as a drug target require further investigation.

14-3-3 proteins are a family of highly conserved proteins abundantly expressed in the brain, accounting for approximately 1% of total soluble brain proteins (Pair and Yacoubian, 2021). YWHAZ encodes the zeta (ζ) isoform of the 14-3-3 protein family (with delta (δ) as its phosphorylated form) (Aitken et al., 1995), which has a complex relationship with AD pathology. *In vitro* studies have found that 14-3-3ζ may negatively regulate Aβ-mediated toxicity (Nelson and Alkon, 2007), and increased levels of insoluble 14-3-3ζ have been detected in NFTs in the hippocampal tissue of AD patients (Umahara et al., 2004). Additionally, 14-3-3ζ maintains cytoskeletal dynamics by inhibiting the binding of phosphorylated cofilin to filamentous actin in AD pathology (Kim et al., 2009; Mizuno, 2013). The expression of 14-3-3ζ in various brain regions of AD patients remains unclear. Some studies have found increased levels of 14-3-3ζ in the cerebrospinal fluid of AD patients, which correlate with elevated phosphorylated Tau 181 (P-Tau) levels (Qiang et al., 2024). Moreover, significant upregulation of 14-3-3ζ has been observed in the frontal and temporal cortices of AD patients (Qureshi et al., 2013), while other studies have reported that the average levels of all 14-3-3 isoforms in the prefrontal cortex of AD patients are lower than those in controls, with no significant difference in the expression level of 14-3-3ζ (Gu et al., 2020). We found that the YWHAZ gene is downregulated in the hippocampal tissue of AD patients, but further research is needed to determine changes in 14-3-3ζ protein levels.

Ubiquitin (Ub) is involved in various cellular pathways, including signal transduction and proteasomal degradation (Hershko and Ciechanover, 1998; Park and Ryu, 2014). UBB is one of the genes encoding polyubiquitin proteins (Wiborg et al., 1985) and contributes significantly to the total ubiquitin protein pool in brain tissue (Ryu et al., 2007). *In vitro* experiments have shown that disruption of the UBB gene leads to reduced self-renewal capacity of neural stem cells, affecting their differentiation into neurons (Park et al., 2020). Animal studies have found that UBB is highly expressed in the mouse olfactory bulb, hippocampus, and hypothalamus (Park et al., 2012). The ubiquitin-proteasome system (UPS) is the primary mechanism for protein quality control within cells (Pohl and Dikic, 2019). The frameshift form of the polyubiquitin protein encoded by the UBB gene, UBB+1, inhibits the ubiquitin-proteasome pathway (Lindsten et al., 2002). UBB+1 is present in the brain tissues of all AD patients (Ebrahimi-Fakhari et al., 2012) and co-localizes with pathological changes in Tau protein, involving the early induction of Aβ deposition and phosphorylated Tau protein aggregation in AD (Hol et al., 2005). UBB+1 also participates in the regulation of gene expression. Transcriptome analysis indicates that low expression of UBB+1 increases the expression of genes involved in ubiquitin-related processes and autophagy pathways (Chen et al., 2021). Therefore, our finding of UBB downregulation in the hippocampus of AD patients may be related to the accumulation of UBB+1 in hippocampal tissue.

α-syn is expressed in presynaptic terminals of neurons (Iwai et al., 1995) and is closely associated with various presynaptic processes (George et al., 1995). Strong mRNA expression of α-syn has been observed in the hippocampus of rats (Maroteaux and Scheller, 1991). α-syn is the main protein component of Lewy bodies, which are hallmarks of Parkinson’s disease (Spillantini et al., 1997). Accumulation of α-syn and Lewy body-related pathology has been found in the brains of more than half of AD patients (Lippa et al., 1998; Hamilton, 2000; Arai et al., 2001). Even in the absence of Lewy body-related pathology, significant increases in intracellular soluble α-syn monomers and oligomers have been observed in the inferior temporal cortex of AD patients (Larson et al., 2012), and elevated α-syn levels have also been detected in the cerebrospinal fluid of AD patients (Twohig and Nielsen, 2019b). Research shows that α-syn induces intracellular Tau aggregation (Spencer et al., 2016) and contributes to Aβ pathology (Masliah et al., 2001), consistent with abundant α-syn in the core of Aβ plaques (Masliah et al., 1996) and the co-localization of α-syn-positive inclusions with neurofibrillary tangles (Arai et al., 2001). Although studies commonly report α-syn accumulation in the central nervous system of AD patients, animal experiments have shown that the loss of endogenous α-syn exacerbates neurodegeneration (Qi et al., 2017). Our study found that SNCA gene expression is downregulated in the hippocampal tissue of AD patients, which may be associated with late-stage AD pathology.

HSPA8 encodes Heat Shock Cognate Protein 70 (HSC70), a molecular chaperone of the heat shock protein (HSP) family (Stricher et al., 2013).HSC70 recognizes substrate proteins in chaperone-mediated autophagy (CMA) (Liao et al., 2021), and the carboxyl-terminus of Hsc70 interacting protein (CHIP) connects to the UPS to target various proteins for degradation (Meimaridou et al., 2009).HSC70 has a complex relationship with AD pathology. Experiments have shown that Tau protein fragments interact with HSC70 and are degraded via the CMA pathway (Wang et al., 2009). Enhancing the binding of HSC70 to Tau protein can significantly reduce Tau levels in cells (Young et al., 2016).In addition to promoting Tau protein degradation, HSC70 is also associated with the neuroprotective effects of soluble APPα (sAPPα) fragments generated by APP cleavage. Increased HSC70 mRNA expression was observed in SH-SY5Y cells treated with sAPPα (Masi et al., 2023).On the other hand, experiments have found that inhibiting HSC70 activity can reduce amyloid deposition and PHF-Tau formation in the CA1 region of AD model mice (Yang and Tohda, 2018). The carboxyl-terminus of Hsc70 interacting protein (CHIP) enhances the ubiquitination and clearance of Neurofilament Medium Chain (NF-M) which may relate to neurodegeneration in AD (Wang et al., 2011). Currently, a significant increase in HSC70 levels has been found in the cytoplasmic and membrane regions of the inferior temporal gyrus in AD patients, accompanied by a decrease in heat shock gactor 1 (HSF-1), suggesting that the elevated HSC70 levels may not be due to increased transcription (Piedrahita et al., 2016). Other studies have found significantly reduced HSPA8 mRNA expression in the hippocampal tissue of AD patients (Silva et al., 2014), which is consistent with the differential expression analysis results of this study.

After analyzing the included studies, we found that various TCM improved behavioral disorders and alleviated hippocampal lesions at different stages of the AD model mice. Curcumin showed efficacy in both early and late stages of AD pathology in APP/PS1 mice, which is due to its effects on Aβ, Tau pathology, and neuroinflammation inhibition. In our target gene analysis of the active components of traditional Chinese medicines, including dihydroartemisinin, berberine, naringin, calycosin, verbascoside, echinacoside, and icariside II, we found that echinacoside involves the ERK and ROS/ATF3/CHOP pathways for its neuroprotective effects. However, the specific targets of echinacoside for AD pathology require further investigation. Other TCM, such as *E. brevicornu* Maxim., *Coptis chinensis* Franch., and *Astragalus propinquus* Schischkin, are important drugs in the current clinical treatment of AD in Chinese medicine, but the majority of their target genes are derived from studies on non-neurological diseases.

Based on our analysis of studies and experiments related to the active components of TCM, we found that berberine (Wu, 2021), icariside II (Yan, 2017), and echinacoside (Dai et al., 2020) can inhibit the phosphorylation of the PERK/eIF2α pathway. The endoplasmic reticulum (ER) maintains cellular protein homeostasis, and the accumulation of unfolded proteins within the cell causes ER stress, which activates the unfolded protein response (UPR) (Uddin et al., 2020). However, ER stress also creates a positive feedback loop with neuroinflammation, leading to neurodegenerative diseases, including AD (Santos and Ferreira, 2018). During ER stress, PERK activation leads to the phosphorylation of eIF2α (Sudhakar et al., 2000), which reduces protein accumulation in the ER by inhibiting mRNA translation (Ohno, 2014). RNA-binding proteins (RBPs) are involved in the formation of stress granules (SGs) (Jackson et al., 2010). Current research has shown a significant increase in phosphorylated PERK and eIF2α in the hippocampal tissues of AD patients (Stutzbach et al., 2013). Long-term overactivation of the PERK/eIF2α pathway leads to reduced protein synthesis, pathological Tau protein phosphorylation, and Aβ production (Moradi Majd et al., 2020). Additionally, the interaction between Tau and SGs results in the formation of insoluble Tau protein aggregates (Vanderweyde et al., 2016). The RNA binding protein (RBP) cascade hypothesis of neurodegeneration proposes that SGs persist for a long time during the pathological process of AD and other diseases (Wolozin and Ivanov, 2019), gradually making proteins like Tau highly stable in their conformations, ultimately leading to neurodegeneration. Among the active components of TCM, berberine (Wu, 2021), icariside II (Yan, 2017), and echinacoside (Dai et al., 2020) were found to reduce the levels of phosphorylated PERK and phosphorylated eIF2α in the hippocampal tissues of APP/PS1 mice. Furthermore, the MicroScale Thermophoresis (MST) Assay demonstrated that echinacoside has a high affinity for human PERK (Dai et al., 2020), suggesting echinacoside’s potential role in regulating protein homeostasis in AD pathology.

This study specifically focused on single-component TCM treatments to assess their effects on hippocampal pathology in AD. Consequently, the reviewed literature may not fully encompass all TCM treatments and formulation patterns used clinically for AD. Additionally, due to the considerable variation in the number of target genes for each active component, we prioritized those with significant overlap with the DEGs for analysis. This approach may have excluded some TCM components with potential therapeutic effects on AD, necessitating further investigation.

To address these limitations, future research on TCM treatments for AD should prioritize identifying and validating the target genes of active components commonly used in clinical treatments through *in vivo* and *in vitro* experiments. Furthermore, animal studies should independently assess the effects of TCM on pathological changes in different brain regions of AD model animals.

In summary, this study conducted a bioinformatic analysis on the screened microarray and high-throughput data, identifying upregulated and downregulated hub genes. Among them, we found that the genes encoding α-syn (SNCA), polyubiquitin (UBB), and mitochondrial outer membrane proteins VDAC (VDAC1, VDAC2) are downregulated hub genes in the hippocampal tissue of AD patients. Based on enriched pathways and hub genes, we found that synaptic function in the hippocampal tissue of AD patients is impaired, associated with structural and functional damage to neuronal mitochondria and disruptions in cellular energy metabolism, consistent with previous findings in several studies on central AD pathology (Swerdlow, 2018; Wang et al., 2014). In the included studies, geniposide (Zhang, 2019), total alkaloids from Dendrobium nobile (Yang, 2017), and tetrahydroxy-stilbene-glucoside (Chen C., 2017) were found to be related to the enriched pathways of DEGs in this study, specifically affecting mitochondrial autophagy and synaptic function in the hippocampal tissue of AD model mice.

Based on target gene analysis, we believe that the active component echinacoside from traditional Chinese medicine has therapeutic potential for AD pathology. Cistanche is a widely used TCM for treating AD. Echinacoside, an active component of Cistanche, has been identified through target gene analysis to interact with specific targets (NTRK1, MAPK1, SNCA, DDIT3) in Parkinson’s disease model cells, which are also DEGs in our study. Furthermore, current research indicates that echinacoside inhibits phosphorylation of the PERK/eIF2α pathway, crucial for maintaining intracellular protein homeostasis. Therefore, we propose that echinacoside holds significant therapeutic potential for addressing AD pathology. However, the therapeutic effects of the aforementioned TCMs on AD require further investigation in future clinical and molecular biology studies.

## Data Availability

The original contributions presented in the study are included in the article/Supplementary Material, further inquiries can be directed to the corresponding author.
