# Supplementary material for: Bioinformatic analysis of hippocampal histopathology in Alzheimer’s disease and the therapeutic effects of active components of traditional Chinese medicine
Source: Front Pharmacol. 2024 Aug 16;15:1424803. doi: 10.3389/fphar.2024.1424803 (PMC11362046; doi:10.3389/fphar.2024.1424803)
Supplement: Supplementary file 3 [file Table7.DOCX]

| Active components of TCM | Gene symbol | UniProtKB/ Swiss-Prot | Protein name |
| --- | --- | --- | --- |
| Dcihydroartemisinin | RPL10 | P27635 | Large ribosomal subunit protein uL16 |
|  | RPL14 | P50914 | Large ribosomal subunit protein eL14 |
|  | RPL23A | P62750 | Large ribosomal subunit protein uL23 |
|  | RPL35 | P42766 | Large ribosomal subunit protein uL29 |
|  | RPS17 | P08708 | Small ribosomal subunit protein eS17 |
|  | RPS19 | P39019 | Small ribosomal subunit protein eS19 |
|  | RPS9 | P46781 | Small ribosomal subunit protein uS4 |
|  | RPL18 | Q07020 | Large ribosomal subunit protein eL18 |
|  | RPL4 | P36578 | Large ribosomal subunit protein uL4 |
|  | ENO1 | P06733 | Alpha-enolase |
|  | LDHA | P00338 | L-lactate dehydrogenase A chain |
|  | PGK1 | P00558 | Phosphoglycerate kinase 1 |
|  | MYC | P01106 | Myc proto-oncogene protein |
|  | HNRNPK | P61978 | Heterogeneous nuclear ribonucleoprotein K |
|  | PPIA | P62937 | Peptidyl-prolyl cis-trans isomerase A |
|  | SOD2 | P04179 | Superoxide dismutase [Mn], mitochondrial |
| Berberine | RELA | Q04206 | Transcription factor p65 |
|  | EGFR | P00533 | Epidermal growth factor receptor |
|  | SIRT1 | Q96EB6 | NAD-dependent protein deacetylase sirtuin-1 |
|  | EP300 | Q09472 | Histone acetyltransferase p300 |
|  | MAPK8 | P45983 | Mitogen-activated protein kinase 8 |
|  | PARP1 | P09874 | Poly [ADP-ribose] polymerase 1 |
|  | FAS | P25445 | Tumor necrosis factor receptor superfamily member 6 |
|  | H3C7 | P68431 | Histone H3.1 |
|  | H3C10 |  |  |
|  | HAT1 | O14929 | Histone acetyltransferase type B catalytic subunit |
| Naringin | FAS | P25445 | Tumor necrosis factor receptor superfamily member 6 |
|  | RELA | Q04206 | Transcription factor p65 |
|  | VCAM1 | P19320 | Vascular cell adhesion protein 1 |
|  | CCL5 | P13501 | C-C motif chemokine 5 |
|  | CYP1B1 | Q16678 | Cytochrome P450 1B1 |
|  | MAPK1 | P28482 | Mitogen-activated protein kinase 1 |
|  | EGFR | P00533 | Epidermal growth factor receptor |
|  | CTNNB1 | P35222 | Catenin beta-1 |
|  | MT3 | P25713 | Metallothionein-3 |
|  | CYBA | P13498 | Cytochrome b-245 light chain |
|  | CYCS | P99999 | Cytochrome c |
|  | CYP1A1 | P04798 | Cytochrome P450 1A1 |
|  | HRAS | P01112 | GTPase HRas |
| Calycosin | PRDX2 | P32119 | Peroxiredoxin-2 |
|  | PRDX1 | Q06830 | Peroxiredoxin-1 |
|  | PGAM1 | P18669 | Phosphoglycerate mutase 1 |
| Verbascoside | ARNT | P27540 | Aryl hydrocarbon receptor nuclear translocator |
|  | RELA | Q04206 | Transcription factor p65 |
|  | EGFR | P00533 | Epidermal growth factor receptor |
|  | MAPK1 | P28482 | Mitogen-activated protein kinase 1 |
|  | SOD2 | P04179 | Superoxide dismutase [Mn], mitochondrial |
|  | SNAI1 | O95863 | Zinc finger protein SNAI1 |
|  | KDM4A | O75164 | Lysine-specific demethylase 4A |
|  | RB1 | P06400 | Retinoblastoma-associated protein |
|  | SMAD2 | Q15796 | Mothers against decapentaplegic homolog 2 |
|  | CDK2 | P24941 | Cyclin-dependent kinase 2 |
| Echinacoside | NTRK1 | P04629 | High affinity nerve growth factor receptor |
|  | MAPK1 | P28482 | Mitogen-activated protein kinase 1 |
|  | SNCA | P37840 | Alpha-synuclein |
|  | DDIT3 | P35638 | DNA damage-inducible transcript 3 protein |
| Icariside II | GOT1 | P17174 | Aspartate aminotransferase, cytoplasmic |
|  | MAPK1 | P28482 | Mitogen-activated protein kinase 1 |
|  | MTOR | P42345 | Serine/threonine-protein kinase mTOR |
|  | PDPK1 | O15530 | 3-phosphoinositide-dependent protein kinase 1 |
|  | BRAF | P15056 | Serine/threonine-protein kinase B-raf |
|  | PARP1 | P09874 | Poly [ADP-ribose] polymerase 1 |
|  | EGFR | P00533 | Epidermal growth factor receptor |
