# Supplementary material for: Bioinformatic analysis of hippocampal histopathology in Alzheimer’s disease and the therapeutic effects of active components of traditional Chinese medicine
Source: Front Pharmacol. 2024 Aug 16;15:1424803. doi: 10.3389/fphar.2024.1424803 (PMC11362046; doi:10.3389/fphar.2024.1424803)
Supplement: Supplementary file 5 [file Table4.DOCX]

| **Author** | **Age** | **Animal model** | | **TCM treatment** | **Administration Route** | **Treatment protocol and grouping** | | **Treatment duration** | **Behavioral experiment** | **Changes in hippocampal tissue indicators and their detection** |
| --- | --- | --- | --- | --- | --- | --- | --- | --- | --- | --- |
|  |  | AD | Control |  |  | **Control** | **AD** |  |  |  |
| Nie, H.  2011[34] | 3-month | APP/PS1 double transgenic mice | Same background APP/PS1 negatively transgenic mice | β-asarone | oral gavage | distilled water：13 | distilled water：13  β-asarone (21. 2mg/kg/d)：13  donepezil (2mg/kg/d)：13 | 4 months | Morris water maze | Amyloid plaques (-) (Congo red dyeing);  LRP-1(+)( immunohistochemistry) |
| Wang, H.  2011[35] | 3-month | APP/PS1 transgenic mice | C57/BL6 mice | curcumin | oral gavage | 0.5% CMC: 12 | 0.5% CMC: 12  rosiglitazone (10mg/kg/d): 12  curcumin (10mg/kg/d): 12  curcumin (20mg/kg/d): 12  curcumin (40mg/kg/d): 12 | 3 months | Morris water maze | GLUT1(+), GLUT3(+)（wb） |
| Wang, P. W  2011[36] | 3-month | APP/PS1 transgenic mice | C57/BL6 mice | curcumin | oral gavage | normal feeding: 12 | 0.5% CMC: 12  rosiglitazone (10mg/kg/d): 12  curcumin (100mg/kg/d): 12  curcumin (200mg/kg/d): 12  curcumin (400mg/kg/d): 12 | 3 months | Morris water maze | Number of synapses; electron microscope images;  Synapse-associated proteins: PSD95(+), IDE(+), NEP(+), PS2(+);  Aβ-related proteins: Aβ40(-), Aβ42(-), ADDLs(-);  Insulin pathway: InR(-), IRS-1(-), p-IRS-1(+), PI3K(+), p-PI3K(+), AKT(+), p-AKT(+) (wb) |
| Wei, P.  2013[37] | 3-month | APP/PS1 transgenic mice | C57/BL6 mice | curcumin | oral gavage | 0.5% CMC: 15 | 0.5% CMC: 15  rosiglitazone (10mg/kg/d): 15  curcumin (100mg/kg/d): 15  curcumin (200mg/kg/d): 15  curcumin (400mg/kg/d): 15 | 6 months | Morris water maze | Number of synapses; electron microscope images;  Shank1(+), PSD95(+) (wb) |
| Dang, H. Z.  2015[38] | 3-month | APP/PS1 transgenic mice | C57/BL6 mice | curcumin | oral gavage | 0.5% CMC: 12 | 0.5% CMC: 12  rosiglitazone (10mg/kg/d): 12  curcumin (100mg/kg/d): 12  curcumin (200mg/kg/d): 12  curcumin (400mg/kg/d): 12 | 6 months | Morris water maze | InR(-), IRS-1(-), p-IRS-1(+)；PI3K(+), p-PI3K(+)；Akt(+), p-Akt(+)；(immunohistochemistry) (wb) |
| Fan, H.  2015[39] | 3-month | APP/PS1 transgenic mice | C57/BL6 mice | curcumin | oral gavage | 0.5% CMC: 15 | 0.5% CMC: 15  rosiglitazone (10mg/kg/d): 15  curcumin (100mg/kg/d): 15  curcumin (200mg/kg/d): 15  curcumin (400mg/kg/d): 15 | 6 months | Morris water maze | Aβ40(-), Aβ42(-), ADDLs(-)（wb） |
| Li, F.  2015[40] | 5-month | APP/PS1 transgenic mice | Wild Type mice | β-asarone | oral gavage | distilled water(0.1ml/10g/d)：13 | distilled water(0.1ml/10g/d)：13  β-asarone(21.2mg/kg/d)：13  donepezil Hydrochloride(2mg/kg/d):13 | 2 months | Morris water maze | Synaptic density (Golgi silver staining)  SYN1(+), PSD95(+), ROCK2(-), LIMK1(-) (wb) |
| Yuan, L.  2016[41] | 5-month | APP/PS1 transgenic mice | C57/BL6 mice | glycosides of Cistanche | intraperitoneal injection | saline: 6 | saline：6  memantine (5 mg/kg): 6  glycosides of Cistanche (200 mg/kg): 6 | 8 weeks | Morris water maze | SOD(+), GSH(+), MDA(-) (ELISA) |
| Chen, C  2017[42] | 4-5 months | APP/PS1 transgenic mice | Wild Type mice | tetrahydroxy stilbene glucoside | oral gavage | distilled water：21  tetrahydroxy stilbene glucoside (100mg/kg/d): 21 | distilled water (0.1ml/10mg/d): 20  tetrahydroxy stilbene glucoside (50mg/kg/d): 20  tetrahydroxy stilbene glucoside (100mg/kg/d): 20  donepezil (1mg/kg/d):19 | 12 months | Morris water maze, Novel Object Recognition, Spontaneous activity experiment | Amyloid plaques (-) (Congo red stain, immunohistochemistry); senile plaques (-) (sulforaphane stain)  APP695(-), BACE-1(-), PS1(-), synaptophysin(+)（wb）；Insoluble Aβ42(-)（ELISA） |
| Chen, Y. W.  2017[43] | 8 months | APP/PS1 transgenic mice | Wild Type mice | curcumin | intraperitoneal injection | normal feeding: 10 | normal feeding: 10  curcumin (150mg/kg/d): 10 | 4 weeks | Morris water maze | IL-1β(-), TNF-α(-)（ELISA） |
| Gao, J.  2017[44] | 7-8 months | APP/PS1 transgenic mice | C57/BL6 mice | calycosin | subcutaneous injection | normal feeding: 12  dimethyl sulfoxide (0.5％; 10ml/kg/d): 12  calycosin (40mg/kg/d) : 12 | normal feeding: 12  calycosin (10mg/kg/d) :12  calycosin (20mg/kg/d) :12  calycosin (40mg/kg/d) :12 | 70 days | Morris water maze, Passive avoidance test | Aβ(-), tau(-), AChE(-), ACh(+), IL-1β(-), TNF-α(-), MDA(-), GSH(+)（ELISA） |
| Yan, L. L  2017[45] | 9 months | APP/PS1 transgenic mice | Wild Type mice | icariside II | oral gavage | saline: 10  icariside II (10mg/kg): 10  icariside II (30mg/kg) :10 | saline: 12  icariside II (10mg/kg): 12  icariside II (30mg/kg) :13 | 3 months | Morris water maze | Neuronal morphology and number (Nissl staining); senile plaque deposits (-) (thioflavin S fluorescence staining);  Aβ1-42(-), Aβ1-40(-)；sAPPα(+), ADAM10(+), APP(-), sAPPβ(-), BACE1(-)；p-eIF2α (-), p-PERK (-), PPARγ(+)；PDE5A(-)（wb） |
| Yang, X. H.  2017[46] | 7 months | APP/PS1 transgenic mice | C57/BL6 mice | Dendrobium nobile Lindl. Alkaloids | oral gavage | saline: 12  Dendrobium nobile Lindl. Alkaloids 40 mg/kg/d: 12 | saline: 12  Dendrobium nobile Lindl. Alkaloids 40 mg/kg/d: 12 | 6 months | Morris water maze | Neuronal morphology (Niehl's stain); senile plaques (-) (sulforaphane S stain); electron micrographs of the hippocampus: synaptic structures;  Aβ1-40(-) , Aβ1-42(-)；GFAP(-)；COX-2(-), IL-6(-)；NF-κB (-), p38(-)；PSD-95(+), SYP(+)（wb） |
| Gu, T.  2018[47] | 3 months | APP/PS1 transgenic mice | C57/BL6 mice | drynariarhizome extract | oral gavage | double distilled water: 15 | double distilled water: 15  drynariarhizome extract (97.5mg/kg/d): 15  donepezil Hydrochloride (0.65mg/kg/d): 15 | 3 months | Morris water maze, Novel Object Recognition | Histomorphology (HE staining), electron micrographs  ERα(+), Erβ(+), FSHR(+), LHR(+)（immunohistochemistry）；p-P38/P38(-)；APP(-), BACE1(-)；P-Tau396/Tau(-), CDK5(-)；Bax(-), caspase-3(-), Bcl(+)（wb）  Aβ(-)；Ach(+), ChAT(+), AchE(-)；IL-1β(-), IL-6(-), TNF-α(-)（ELISA） |
| Liu, J. C.  2018[48] | 3 months | APP/PS1 transgenic mice | C57/BL6 mice | psoralen extract | oral gavage | double distilled water: 15 | double distilled water: 15  psoralen extract (0.5g/kg/d): 15  donepezil Hydrochloride (0.65mg/kg/d): 15 | 3 months | Morris water maze, Novel Object Recognition, Dark avoidance test | electron micrographs,  Erα(+), Erβ(+), FSHR(+), LHR(+)（immunohistochemistry）；P-ERK/ERK(+), Aβ1-42(-), APP(-), BACE1(-), pTau396(-), Cdk5(-), Ach(+), AchE(-), ChAT(+)； IL-1(-), IL-6(-), TNF-α(-), Bad(-), Bcl-2(+), NF-kB(-), Caspase-3(-)（wb） |
| Yu, X. F.  2018[49] | 3 months | APP/PS1 transgenic mice | C57/BL6 mice | eucommia ulmoides extract | oral gavage | saline: 15 | double distilled water: 15  psoralen extract (1.3g/kg/d): 15  donepezil Hydrochloride (1mg/kg/d): 15 | 3 months | Morris water maze, Novel Object Recognition, Dark avoidance test | HE staining, electron microscope picture;  Erα(+), Erβ(+), FSHR(+), LHR(+)（immunohistochemistry）；Aβ1-42(-), APP(-), BACE1(-), Ach(+), ChAT(+), AchE(-), Erβ(+), p-JNK(-), pTau396(-), GSK-3β(-), Cdk5(-), NMDAR1(+), GluR2(+), CaMK II(-), Bcl-2(+), Bax(-), Caspase-3(-)（wb） |
| Liu, S. W.  2019[50] | 6 months | APP/PS1 transgenic mice | C57/BL6 mice | Valerenicacid, MPEG-PLA-SS-VA NPs | intravenous Injections | saline: 10 | saline: 10  Blank NPs: 10  Valerenicacid: 10  MPEG-PLA-SS-VA NPs: 10 | 8 weeks | Morris water maze, Novel Object Recognition, | Apoptosis status (Tunel staining)  Aβ (-), number of GFAP positive cells (-), number of Iba-1 positive cells (-) (immunofluorescence)  SOD(-), GSH-Px(-), CAT(-), MDA(+)；IL-1β(-), TNF-α(-), IL-4(+), IL-10(+)（ELISA）  GLUT1(+), LRP1 mRNA(+), NF-κB mRNA (-)（Rt-PCR）  GLUT1(+), LRP1(+), NF-Κb(-)（wb） |
| Piao, Z.Y.  2019[51] | 6 months | APP/PS1 transgenic mice | C57/BL6J mice | schisandrin | oral gavage | distilled water：10 | distilled water：17  schisandrin + 0.5%CMC: 18 | 30 days | Morris water maze | Nysted staining of hippocampal tissue;  RAGE mRNA(-)（RT-PCR）；RAGE(-), p-P38(-)（wb） |
| Yang, S. S.  2019[52] | 6 months | APP/PS1 transgenic mice | C57/BL6 mice | smilagenin | oral gavage | 0..5% CMC: 10  smilagenin (26 mg/kg/d):10 | 0..5% CMC: 10  smilagenin (26 mg/kg/d):10 | 2 months | Mirror water maze, Open field test, Y maze, Novel object recognition | Amyloid plaque (-) (Thioflavin S staining)  BDNF mRNA (+) (RT-PCR)  Histone H3ac (+), P300 protein (+) |
| Zhang, Z. H.  2019[53] | 6 months | APP/PS1 transgenic mice | C57/BL6 mice | geniposide | oral gavage | equal volume of water: 15 | similar volume of water: 15  geniposide (50mg/kg/d): 15 | 6 weeks | Mirror water maze, Open field test. | HE staining; hippocampal SPs plaques (-)  soluble Aβ1-40 (-), soluble Aβ1-42 (-) (ELISA)  LC3-II (+), Beclin1 (+), p62 (-), p-Akt/Akt (-), p-mTOR/mTOR (-), p-4E-BP1/4E-BP1 (+) (WB)  Akt mRNA (-), mTOR mRNA (-), 4E-BP1 mRNA (+) (RT-PCR) |
| Zhu, T. R.  2019[54] | 4 months | APP/PS1 transgenic mice | C57/BL6 mice | icariin | oral gavage | distilled water：8  icariin: 8 | distilled water：8  icariin: 8 | 8 months | Morris water maze | insoluble Aβ40(-),insoluble Aβ42(-), soluble Aβ40(-)（ELISA） |
| Kong, F. G.  2020[55] | 8 months | APP/PS1 transgenic mice | Wild Type mice | forsythoside B | oral gavage | saline (5ml/kg/d): 12 | saline (5ml/kg/d): 12  forsythoside B (10mg/kg/d): 12  forsythoside B (40mg/kg/d): 12 | 4 weeks | Mirror water maze, Open field test, Y maze | Fibrillar amyloid plaques of Aβ (-) (Thioflavin S staining); phosphorylated Tau protein (-); 4-HNE (-); GFAP (-) (immunohistochemistry) |
| Wang, R. R.  2020[56] | 4 months | APP/PS1 transgenic mice | C57/BL6 mice | icariin | oral gavage | equal volume of distilled water: 6 | similar volume of distilled water: 6  icariin (60mg/kg/d): 6 | 8 months | Morris water maze | Iba-1(-), GFAP(-), Number of IL-1β (-) staining positive regions (immunohistochemistry)；Bcl-2(+), Bax(-)；p-p65/p65(-)；nlrp3(-), caspase1(-)（wb） |
| Kong, Y. Y.  2020[57] | 6 months | APP[K670N/M671L(Swedish)+ I716V(Florida)+  V717I(London)]+ PS1[M146L+ L286V] （5×FAD） | Wild Type mice | paeoniflorin | intraperitoneal injection | equal volume of saline: 15 | paeoniflorin (5 mg/kg): 30  similar volume of saline: 15 | 28 days | Morris water maze, T-maze test | Aβ (-)（immunofluorescence） |
| Wu, Y.  2021[58] | 6 months | APP/PS1 transgenic mice | C57/BL6 mice | berberine | Oral administration via feed | normal feeding: 10  berberine (260mg/kg): 15 | normal feeding: 10  berberine (260mg/kg): 15 | 3 months | Morris water maze | Electron micrograph;Aβ42(-)（ELISA）；APP(-), BACE1(-), CTF99(-), Bip (-), p-PERK/PERK(-), p-eIF2α/eIF2α(-), tau ps202(-), tau ps404(-), p-GSK-3β Y216/GSK-3β(-)（wb） |
| Wang, C. Y.  2022[59] | 8 months | APP/PS1 transgenic mice | Wild Type mice | forsythoside A | oral gavage | saline (10ml/kg/d): 12 | saline (10ml/kg/d): 12  forsythoside A(30mg/kg/d): 12 | 28 days | Mirror water maze, Open field test, Y maze | Tissue morphology (HE staining), neuronal apoptosis (-) (TUNEL staining); Aβ (-), tau (-); Iba1 (-), GFAP (-) (immunohistochemistry). |
| Zhao, D. P.  2022[60] | 4 months | APP/PS1 transgenic mice | B6C3F1 mice | Rehmannia glutinosa granules | oral gavage | saline: 7 | saline: 13  Rehmannia glutinosa granules (5g/kg/d) (1000mg/ml): 14  Rehmannia glutinosa granules (12g/kg/d) (2500mg/ml): 13 | 3 months | Morris water maze, Nesting test, Rotarod test, Conditioned fear test | senile plaques (-) (Thioflavin T staining);  CD34 (+), fibrinogen (-) (immunofluorescence); Occludin (+), ZO-1 (+) (WB) |
| He, J.  2023[61] | 6 months | APP/PS1 transgenic mice | Wild Type mice | dihydroartemisinin | oral gavage | dimethyl sulfoxide: 15  dihydroartemisinin (20mg/kg/d): 15 | dimethyl sulfoxide: 15  dihydroartemisinin (20mg/kg/d): 15 | 3 months | Morris water maze, Open field test, Elevated plus maze test | Aβ1-40(-)；LPS(-), MCP-1(-), TNF-α(-), IL-1β(-)（immunofluorescence）；APP(-), BACE1(-), Iba-1 (-), GFAP(-)（immunofluorescence）（wb）；ZO-1(+), Occludin(+), Claudin5(+)；TLR4(-), MyD88(-), p-IκB-α(-),  NF-κB(-), IκB-α(+)（wb） |
| Li, X.  2016[62] | 4 months | APP/PS1 transgenic mice | Wild Type mice | RAPO-1-3, Onjisaponin B | oral gavage | 200 μl of vehicle：12-16 | 200 μl of Onjisaponin B (1 mg/ml)：12-16  200 μl of RAPO-1-3 (15 mg/ml) ：12-16  200 μl of vehicle：12-16  per 20 g body weight | 3 months | Morris Water Maze Test | SDS-soluble and FA-soluble Aβ40(-)（ELISA） |
| Shi, X. M.  2018[63] | 9 months | APP/PS1 transgenic mice | C57/BL6 mice | Safflower (yellow hydroxyl safflower yellow A) (safflower yellow B) | oral gavage | the same volume of normal saline：10 | the same volume of normal saline：8  Safflower yellow 10 mg/kg：8  Safflower yellow 30 mg/kg：8  Safflower yellow 100 mg/kg：8  galanthamine hydrobromide 3 mg/kg：8  0.01 ml/g body weight | 90 days | Morris Water Maze Test；Step-down test | neuronal loss and death in hippocampus：CA1, CA3(-)； activated astrocytes in hippocampus CA1, CA3, DG region(-)；（HE staining）  Aβ accumulation in hippocampus：CA1, CA3, DG region(-)；soluble and insoluble Aβ1-42 in hippocampus(-)（Immunohistochemistry）；BACE1(-), APP(-), sAPPα(+), IDE(+)（wb） |
| Jiang, X. W.  2020[64] | 6 months | APP/PS1 transgenic mice | Wild Type mice | N. incisum ethanol extract | oral gavage | equal volume of deionized water：5 | N. incisum ethanol extract 1.3 g/kg：9  equal volume of deionized water：6 | 2 months | Morris Water Maze | the Aβ40 and Aβ42 levels in the hippocampus(-)（ELISA） |
| Wang, X. F.  2021[65] | 9 months | APP/PS1 transgenic mice | Wild Type mice | 3,6′-disinapoyl sucrose | oral gavage | 0.3% CMC-Na as: 10 | 0.3% CMC-Na as: 10  3,6′-disinapoyl sucrose (5mg/kg): 10  3,6′-disinapoyl sucrose (10mg/kg): 10  3,6′-disinapoyl sucrose (20mg/kg): 10 | 28 days | Morris Water Maze | number of Nissl bodies in CA1, CA3, DG(+)（Nissl staining assay）  Sox-2-positive cells(+), Nestin-positive cells(+)；number of mature neurons (NeuN+/DAPI) in CA1, CA3, DG (+)​（IF staining assay ） |
| Zhou, W. Q.  2009[66] | 6 months | SAMP8 mice | SAMR1 mice | icariin | oral gavage | equal volume of saline: 10 | equal volume of saline: 9  icariin (75mg/kg/d): 10  icariin (150mg/kg/d): 9  donepezil hydrochloride tablets 1mg/kg: 9  diethylstilbestrol 0.2mg/kg: 9 | 12 weeks | Morris Water Maze, Step-down test | Ach (+), MCBC binding capacity (+) (ELISA); DA (+), DOPAC (+), HVA (+) (HPLC); 5-HIAA (+), NE (+) (HPLC); Asp (+), Glu (-), Gln (-) (HPLC-ECD); GABA (+) (HPLC-ECD); Morphology and number of GFAP-expressing astrocytes (-) (immunohistochemistry) |
| Zhang, Z.W.  2015[67] | 6 months | SAMP8 mice | SAMR1 mice | icariin | oral gavage | equal volume of double distilled water(1ml/d): 15 | equal volume of double distilled water(1ml/d): 15  icariin 0.5%CMC (0.01ml/g/d): 10 | 8 weeks | Morris Water Maze | BDNF(+)（Immunohistochemistry）（wb） |
| Chen, W. K.  2019[68] | 75-85 days | SAMP8 mice | SAMR1 mice | 1-Deoxynojirimycin | oral gavage | distilled water: | distilled water  1-Deoxynojirimycin (40mg/kg/d)  1-Deoxynojirimycin (160mg/kg/d)  metformin hydrochloride | 2 months | Morris Water Maze | microglia expression levels, Aβ(-) (immunohistochemistry); TNF-α(-), IL-1β(-), IL-6(-)（ELISA）；BACE1(-), BDNF(+), TrkB(+)（wb） |

**References**

1. Nie, H. (2011). Effect of Active Components in Acorus Gramineus on Learning and Memory and the Neuroprotective Pathway of Clusterin in APP/PS1 Double Transgenic Mice [Doctoral dissertation, Guangzhou University of Chinese Medicine]. <https://kns.cnki.net/kcms2/article/abstract?v=8WLnD7pOpNHHOArH7fC1jf5dZOSzSgKCVT0tBKFe_DkFdnGmfYb17EcdB6QV2VkDJVJ3hfngmggenw-JUY8LtUIVVFlW359MBPibwFeVXtqx4YcBBRQ7hYorQUsckUyanmlApeLBjzY64s7QrcvoNA==>.
2. Wang, H. (2011). The effect of curcumin on brain energy metabolism in APP/PS1 double transgenic mice (Master's thesis, Beijing University of Chinese Medicine). [Master's thesis].<https://kns.cnki.net/kcms2/article/abstract?v=8WLnD7pOpNEeoy5QidlrwcQkc4yvRVKYNOt1z_33-GW1mkBnY0oOPeCI8p3nicp9qnJ83dNZ4JIOOxgxeO0teqcojVBqQzU0jiM5mGCoqOiviQDWko7KbEXDUdR97GcpRdRoNN8_L63sz7OTkl6mtQ==uniplatform=NZKPTlanguage=CHS>
3. Wang, P. W. (2011). The neuroprotective effect of curcumin on APP/PS1 double transgenic mice (Doctoral thesis, Beijing University of Chinese Medicine). [Doctoral thesis].<https://kns.cnki.net/kcms2/article/abstract?v=8WLnD7pOpNG-cYV_5dpx_vC7ofdB9awshvp0pJ3w1Uf2ogAY-TX7w36Ynbme6dY8OW1E457s3CYviPlOoJSUXY6Z-ZF_ejG4hx61NWx3C4r6vq5QDGLHiPW_kDDAdwWq83dVMXo0_zxOAHxZ72OrwQ==uniplatform=NZKPTlanguage=CHS>
4. Wei, P. (2013). The effect of curcumin on the expression of Shank1 and PSD95 in AD mice (Master's thesis, Beijing University of Chinese Medicine). [Master's thesis]. <https://kns.cnki.net/kcms2/article/abstract?v=8WLnD7pOpNG-ZZylnudvM-wQIxmLNBiKDtmekqPyu5tcaAMqqIBR0RTAathYSF-eMjUG72redl6Nnu8xPuscTrhMjdGvOySHIha3oxWqUtS93CPL60bgUpayooPKIQesCkk3JA8rq3D5aqd0abEdnw==uniplatform=NZKPTlanguage=CHS>
5. Dang, H. Z. (2015). The effect of curcumin on the insulin signaling pathway in APP/PS1 double transgenic mice (Doctoral thesis, Beijing University of Chinese Medicine). [Doctoral thesis]. <https://kns.cnki.net/kcms2/article/abstract?v=8WLnD7pOpNHmkM16CMZa-bUn2_N-7slPefZGdktOLUtiV4UQdiSvORprPnHCcldoHhSG-qiHHWnxPdU2oKiqeCYa0KI1JsXIm9O-4lM7C52rxtcC7sstH-3Ny1F2xTyExovzlRrxy4zDnBJZX_WbZQ==uniplatform=NZKPTlanguage=CHS>
6. Fan, H. (2015). The effect of curcumin on Aβ and ADDLs in APPswe/PS1dE9 double transgenic mice (Master's thesis, Beijing University of Chinese Medicine). [Master's thesis].<https://kns.cnki.net/kcms2/article/abstract?v=8WLnD7pOpNFN3ZjQ8_33-SwigUMBnZz4OuB27jdMNW6ErtSS8pMHg-U-EHNq4bpxGcELA3-KzuG08QWM05n-o30hpiP5ghb3941K_7jziPSp8yT3CPdZgmz9LvZ2o08AgKhZrBGrz1N5EcPhVj342A==uniplatform=NZKPTlanguage=CHS>
7. Li, F. (2015). The molecular mechanism of β-asarone against synaptic injury in Alzheimer's disease via the ROCK pathway (Doctoral thesis, Guangzhou University of Chinese Medicine). [Doctoral thesis].<https://kns.cnki.net/kcms2/article/abstract?v=8WLnD7pOpNEB8AdalDwzZETrgU-YGM_vrlogtoHi5taLDgMtIevOZlLNz9uLJtL5toW5lnbBSRykMoxDIUhENLc30VlWRUosqEBfpCWa_Xeq8y8fBLm_quW6_WF4XhyFPHG9Me6im4DP15HBAwsUqA==uniplatform=NZKPTlanguage=CHS>
8. Yuan, L., 2016. Age-related changes in oxidative/antioxidative properties and neurotrophic factor expression in the hippocampus of mice; The effects of traditional Chinese medicine components on learning and memory ability, oxidative/antioxidative balance in APP/PS1 transgenic mice [Dissertation]. Chongqing Medical University, Chongqing. https://doi.org/10.7666/d.D01120429.
9. Chen, C. (2017). Study on the effects of stilbene glycosides on APP/PS1 double transgenic mice and its pharmacological mechanism (Master's thesis, Capital Medical University). [Master's thesis].<https://kns.cnki.net/kcms2/article/abstract?v=8WLnD7pOpNFbrTUp2VQKpahPFGeYUeOSFjYwMd315CMk_C9ZXfKEn2VT_2twzZxsYloGz5dxUEqtC82Qku1LcYsytOKKXdIDnvYck4v81Zv60zZLATF1NIn6Qej2JJdCQZD_MZkFxJwX0-_rUkS1qQ==uniplatform=NZKPTlanguage=CHS>
10. Chen, Y. W. (2017). The effect of curcumin on behavior and inflammation mechanism in APP/PS1 double transgenic mice (Master's thesis, Beijing University of Chinese Medicine). [Master's thesis].<https://kns.cnki.net/kcms2/article/abstract?v=8WLnD7pOpNHsCH3NFRwsQmoD1VQqgeYsZ6qaKmorr0NJN7OUqYJSOSSQ3qeTcYA7oMXfycvCkMj7ObH1NgDS85dd33Z1ffiayfCCtOX1LLKMPXBplBRgBA7PPzmpk5TBmLySIzdvNkZr96wU7Bipag==uniplatform=NZKPTlanguage=CHS>
11. Gao, J. (2017). The mechanism of puerarin in alleviating cognitive impairment in APP/PS1 mice through the PKC/Nrf2 pathway (Doctoral thesis, Jilin University). [Doctoral thesis]. <https://kns.cnki.net/kcms2/article/abstract?v=8WLnD7pOpNGJDJ-jzmHUrUy-sSjP9bAV4l89i2f9oOUMqT7NkanywLgyoNzxjNosGc2ljhA9TCiSfgDCvP6z4LqFLCtVkwpT56-McHtCUtW6WFCcDS0EY-nCtAOf3-e0weKxMBZPlMx62eFjv96kLA==uniplatform=NZKPTlanguage=CHS>
12. Yan, L. L. (2017). Study on the effects of icariin on learning and memory impairment in Alzheimer's disease model mice and its mechanism (Master's thesis, Zunyi Medical College). [Master's thesis].<https://kns.cnki.net/kcms2/article/abstract?v=8WLnD7pOpNGKTBP1WejHd6nG0L8u74lBKmLMEnEzwgamzBuYzIIt8HWspnGbtAQDh47kmoatAwhphV4NzXnvh8KaPHSHmQnjBY0K_UlElyN6UK7tSg0POiQKlUMY4ChNxnYP3GiHva3riSKVllinKw==uniplatform=NZKPTlanguage=CHS>
13. Yang, X. H. (2017). Study on the effect of Dendrobium nobile alkaloids on learning and memory of APP/PS1 transgenic mice and related factors (Master's thesis, Zunyi Medical College). [Master's thesis].<https://kns.cnki.net/kcms2/article/abstract?v=8WLnD7pOpNEC6EvftGNEV3VDt5fRemtteWUGw8BswoRN_XjrUO5PiI7XzDlM8W2P5ofO6bePmqGlOJrShYlgh8a4XdI1iPj36FnNhsZ4cueWBjTeqS3bajMnTMUNtQBuLcgGoQ6ib5uRIAV37Z4_-w==uniplatform=NZKPTlanguage=CHS>
14. Gu, T. (2018). Study on the protective effect and mechanism of Bone Repair Extract on APP/PS1 double transgenic AD model mice and Aβ-damaged PC12 cells (Master's thesis, Heilongjiang University of Chinese Medicine). [Master's thesis]. <https://link.cnki.net/doi/10.27127/d.cnki.ghlzu.2018.000105doi:10.27127/d.cnki.ghlzu.2018.000105>.
15. Liu, J. C. (2018). Study on the protective effect and mechanism of Psoralea Corylifolia extract on APP/PS1 transgenic AD model mice and Aβ-damaged PC12 cells (Master's thesis, Heilongjiang University of Chinese Medicine). [Master's thesis]. <https://link.cnki.net/doi/10.27127/d.cnki.ghlzu.2018.000051doi:10.27127/d.cnki.ghlzu.2018.000051>.
16. Yu, X. F. (2018). Study on the protective effect and mechanism of Eucommia Extract on APP/PS1 transgenic AD model mice and Aβ-damaged PC12 cells (Master's thesis, Heilongjiang University of Chinese Medicine). [Master's thesis].<https://link.cnki.net/doi/10.27127/d.cnki.ghlzu.2018.000065doi:10.27127/d.cnki.ghlzu.2018.000065>.
17. Liu, S. W. (2019). Study on the mechanism of Valerenic Acid monomer of traditional Chinese medicine in Alzheimer's disease (Doctoral thesis, Changchun University of Chinese Medicine). [Doctoral thesis].<https://link.cnki.net/doi/10.26980/d.cnki.gcczc.2019.000022doi:10.26980/d.cnki.gcczc.2019.000022>.
18. Piao, Z.-Y., Song, L., Yao, L.-F., et al., 2019. Effect of schisandrin on learning and memory abilities and their mechanism in APP/PS1 dual

transgenic dementia mice [Journal]. Chinese Journal of Neuromedicine, 18(4), 325-330. https://doi.org/10.3760/cma.j.issn.1671-8925.2019.04.001.

1. Yang, S. S. (2019). Study on the acetylation mechanism of Saponin treatment in Alzheimer's disease (Master's thesis, Shanghai Jiao Tong University). [Master's thesis]. <https://link.cnki.net/doi/10.27307/d.cnki.gsjtu.2019.004603doi:10.27307/d.cnki.gsjtu.2019.004603>.
2. Zhang, Z. H. (2019). Study on the protective effect of Genipin on APP/PS1 mice behavior damage and pathological changes—suppressing mTOR signaling pathway to enhance autophagy (Doctoral thesis, Shanxi Medical University). [Doctoral thesis].<https://kns.cnki.net/kcms2/article/abstract?v=8WLnD7pOpNHTaExJYB65R6cRduLEudLxPFrQd14MyV7-kJGLq1faIO1lw7cA8zY1Sr87UKLn7J264IYLln-IXo6N-mxHLtGkxhOK2SkyZtq6VBY5I-Wo1HApYA-sH1PAZ5Wd7TBBCDdpGEeKL8P3pg==uniplatform=NZKPTlanguage=CHS>
3. Zhu, T. R. (2019). Study on the effect of Icariin on lymphocyte-mediated immune-inflammatory response in Alzheimer's disease (Doctoral thesis, Shandong University). [Doctoral thesis].<https://link.cnki.net/doi/10.27272/d.cnki.gshdu.2019.000112doi:10.27272/d.cnki.gshdu.2019.000112>.
4. Kong, F. G. (2020). The protective effect and mechanism of Forsythoside B on Alzheimer's disease (Master's thesis, Jilin University). [Master's thesis].<https://link.cnki.net/doi/10.27162/d.cnki.gjlin.2020.002010doi:10.27162/d.cnki.gjlin.2020.002010>.
5. WANG Ranran, ZHU Tianrui, ZHANG Feng, WANG Min, MIN Aoxue, LI Heng, LI Xiaohong. Effects of long-term icariin treatment on neuroinflammation in APP/PS1 mice[J]. Journal of Shandong University (Health Sciences), 2020, 58(4): 71-77.
6. Kong, Y. Y. (2020). Study on the protective effect and mechanism of paeoniflorin in transgenic mice with Alzheimer's disease (Master's thesis, Guangdong Pharmaceutical University). [Master's thesis]. <https://link.cnki.net/doi/10.27690/d.cnki.ggdyk.2020.000118doi:10.27690/d.cnki.ggdyk.2020.000118>.
7. Wu, Y. (2021). Study on the molecular mechanism of berberine affecting the cognitive function of APP/PS1 mice (Doctoral thesis, Huazhong University of Science and Technology). [Doctoral thesis]. <https://link.cnki.net/doi/10.27157/d.cnki.ghzku.2021.006532doi:10.27157/d.cnki.ghzku.2021.006532>.
8. Wang, C. Y. (2022). Study on the effect of Forsythoside A on alleviating Alzheimer's disease through the Nrf2/GPX4 pathway (Doctoral thesis, Jilin University). [Doctoral thesis]. <https://link.cnki.net/doi/10.27162/d.cnki.gjlin.2022.000193doi:10.27162/d.cnki.gjlin.2022.000193>.
9. Zhao, D. P. (2022). Study on the protective effect of Rehmannia on blood-brain barrier in APP/PS1 mice and the clinical application of Chinese medicine formula with Rehmannia as monarch medicine in Alzheimer's disease (Doctoral thesis, Nanjing University of Chinese Medicine). [Doctoral thesis]. <https://link.cnki.net/doi/10.27253/d.cnki.gnjzu.2022.000683doi:10.27253/d.cnki.gnjzu.2022.000683>.
10. He, J. (2023). Study on the molecular mechanism of dihydroartemisinin improving cognitive impairment in Alzheimer's disease based on the "gut microbiota-gut-brain axis" (Master's thesis, Chongqing Medical University). [Master's thesis].<https://link.cnki.net/doi/10.27674/d.cnki.gcyku.2023.000092doi:10.27674/d.cnki.gcyku.2023.000092>.
11. Li X, Cui J, Yu Y, Li W, Hou Y, Wang X, Qin D, Zhao C, Yao X, Zhao J, Pei G. Traditional Chinese Nootropic Medicine Radix Polygalae and Its Active Constituent Onjisaponin B Reduce β-Amyloid Production and Improve Cognitive Impairments. PLoS One. 2016 Mar 8;11(3):e0151147. doi: 10.1371/journal.pone.0151147. PMID: 26954017; PMCID: PMC4782990.
12. Shi XM, Zhang H, Zhou ZJ, Ruan YY, Pang J, Zhang L, Zhai W, Hu YL. Effects of safflower yellow on beta-amyloid deposition and activation of astrocytes in the brain of APP/PS1 transgenic mice. Biomed Pharmacother. 2018 Feb;98:553-565. doi: 10.1016/j.biopha.2017.12.099. Epub 2017 Dec 27. PMID: 29288971.
13. Jiang XW, Liu WW, Wu YT, Wu Q, Lu HY, Xu ZH, Gao HY, Zhao QC. Notopterygium incisum extract (NRE) rescues cognitive deficits in APP/PS1 Alzhneimer's disease mice by attenuating amyloid-beta, tau, and neuroinflammation pathology. J Ethnopharmacol. 2020 Mar 1;249:112433. doi: 10.1016/j.jep.2019.112433. Epub 2019 Nov 27. PMID: 31783135.
14. Wang XF, Xiao HH, Wu YT, Kong L, Chen JC, Yang JX, Hu XL. Active constituent of Polygala tenuifolia attenuates cognitive deficits by rescuing hippocampal neurogenesis in APP/PS1 transgenic mice. BMC Complement Med Ther. 2021 Oct 25;21(1):267. doi: 10.1186/s12906-021-03437-5. PMID: 34696749; PMCID: PMC8543956.
15. Zhang, Z.-W., Zhang, T., Zhu, H., 2015. Icarin upregulates brain-derived neurotrophic factors in the hippocampus of the senescence-acceler-ated mouse [Journal]. Chinese Journal of Clinical Physicians, 17(2), 178-182. https://doi.org/10.3760/cma.j.issn.1008-1372.2015.02.007.
16. Zhou, W. Q. (2009). Study on the effect of icariin on the learning and memory of rapidly aging SAMP8 mice and its mechanism (Master's thesis, Chinese Academy of Medical Sciences). [Master's thesis]. <https://kns.cnki.net/kcms2/article/abstract?v=8WLnD7pOpNFTeDh3Bh1ET6AsOpyiyvKYQGtDMKWlohnr0eR4sJ56gCdxp6zRENVUOdE8reoyQeGg-fCPWAvm6VqvmfdNpXZoJGzyVfQANifw2G9obs15LYRZtVQwONt_KHgxODOcjLoFBmeQh_4C4w==uniplatform=NZKPTlanguage=CHS>
17. Chen, W. K. (2019). Study on the neuroprotective effect and mechanism of 1-deoxy-myo-inositol in Alzheimer's disease (Master's thesis, Nanjing University of Chinese Medicine). [Master's thesis].<https://kns.cnki.net/kcms2/article/abstract?v=8WLnD7pOpNGnirnI1ZB2jUdNUO0gKXxXe1Aaq7W54e8rVYDcoDuKicLvNXV65c8MRggMljg8mbcsZKn8eu2ACCUcOC-tER27jG5KAgirmUzc83o7EI7lMHiJUKfsTwWbbqkpN0f1Y71hBDBGz74lug==uniplatform=NZKPTlanguage=CHS>
