# Supplementary material for: Bioinformatic analysis of hippocampal histopathology in Alzheimer’s disease and the therapeutic effects of active components of traditional Chinese medicine
Source: Front Pharmacol. 2024 Aug 16;15:1424803. doi: 10.3389/fphar.2024.1424803 (PMC11362046; doi:10.3389/fphar.2024.1424803)
Supplement: Supplementary file 6 [file Table6.DOCX]

| **Traditional Chinese Medicine** | | [**www.theplantlist.org**](http://www.theplantlist.org)  (accepted name) | **Active components of TCM** | | |
| --- | --- | --- | --- | --- | --- |
| **Chinese** | **Latin name according to**  **pharmacopoeia and included studies** |  | **Chinese** | **English** | **CAS numbers** |
| 石菖蒲 | *Acorus tatarinowii* Schott. | *Acorus calamus* var. angustatus Besser | β-细辛醚 | β-asarone | 5273-86-9 |
| 姜黄 | *Curcuma longa* L. | *Curcuma longa* L. | 姜黄素 | Curcumin | 458-37-7 |
| 何首乌 | *Polygonum multiflorum* Thunb. | *Reynoutria multiflora* (Thunb.) Moldenke | 二苯乙烯苷 | Tetrahydroxy-stilbene-glucoside | 82373-94-2 |
| 黄芪 | *Astragalus membranaceus*(Fisch.) Bge.var.*mongholicus* (Bge.)Hsiao | *Astragalus propinquus* Schischkin | 毛蕊异黄酮 | Calycosin | 20575-57-9 |
|  | *Astragalus membranaceus* (Fisch.) Bge. |  |  |  |  |
| 淫羊藿 | *Epimedium brevicornu* Maxim. | *Epimedium brevicornu* Maxim. | 淫羊藿次苷Ⅱ | Icariside II | 113558-15-9 |
|  | *Epimedium sagittatum* (Sieb.et Zucc.) Maxim. | *Epimedium sagittatum* (Sieb.et Zucc.) Maxim. |  |  |  |
|  | *Epimedium pubescens* Maxim. | *Epimedium pubescens* Maxim. |  |  |  |
|  | *Epimedium koreanum*Nakai. | *Epimedium koreanum*Nakai. | 淫羊藿苷 | Icariin | 489-32-7 |
| 桑叶 | *Morus alba* L. | *Morus alba* L. | 脱氧野尻霉素 | 1-deoxynojirimycin | 19130-96-2 |
| 鸭跖草 | *Commelina communis* L. | *Commelina communis* L. |  |  |  |
| 缬草 | *Valeriana officinalis* L. | *Valeriana officinalis* L. | 缬草烯酸 | Valganciclovir | 175865-60-8 |
| 五味子 | *Schisandra chinensis* (Turcz.) Baill. | *Schisandra chinensis* (Turcz.) Baill. | 五味子醇甲 | Schisandrin | 7432-28-2 |
| 知母 | *Anemarrhena asphodeloides* Bge. | *Anemarrhena asphodeloides* Bge. | 异菝葜皂苷元 | smilagenin | 126-18-1 |
| 栀子 | *Gardenia jasminoides* Ellis | *Gardenia jasminoides* J.Ellis | 京尼平苷 | Geniposide | 24512-63-8 |
| 连翘 | *Forsythia suspensa* (Thunb.) Vahl | *Forsythia suspensa* (Thunb.) Vahl | 连翘酯苷A | Forsythoside A | 79916-77-1 |
|  |  |  | 连翘酯苷B | Forsythoside B | 81525-13-5 |
| 黄连 | *Coptis chinensis* Franch. | *Coptis chinensis* Franch. | 小檗碱 | Berberine | 2086-83-1 |
|  | *Coptis deltoidea* C.Y.Cheng et Hsiao | *Coptis deltoidea* C.Y.Cheng & P.K.Hsiao |  |  |  |
|  | *Coptis teeta* Wall. | *Coptis teeta* Wall. |  |  |  |
| 青蒿 | *Artemisia annua* L. | *Artemisia annua* L. | 双氢青蒿素 | Dihydroartemisinin | 71939-50-9 |
| 芍药 | *Paeonia lactiflora* Pall. | *Paeonia lactiflora* Pall. | 芍药苷 | paeoniflorin | 23180-57-6 |
|  | *Paeonia veitchii* Lynch | *Paeonia anomala* subsp. veitchii (Lynch) D.Y.Hong & K.Y.Pan |  |  |  |
| 红花 | *Carthamus tinctorius* L. | *Carthamus tinctorius* L. | 羟基红花黄色素A | Hydroxyl safflower yellow A | 78281-02-4 |
|  |  |  | 红花黄B | Safflower yellow B | 91574-92-4 |
| 肉苁蓉 | *Cistanche deserticola* Y.C.Ma | *Cistanche deserticola* Y.C.Ma | 松果菊苷 | Echinacoside | 82854-37-3 |
|  | *Cistanche tubulosa* (Schenk) Wight | *Cistanche tubulosa* (Schenk) Wight | 毛蕊花糖苷 | Verbascoside | 61276-17-3 |
| 金钗石斛 | *Dendrobium nobile* Lindl. | *Dendrobium nobile* Lindl. | 石斛碱 | (-)-Dendrobine | 2115-91-5 |
| 骨碎补 | *Drynaria fortunei* (Kunze) J.Sm. | *Drynaria roosii* Nakaike | 新北美圣草苷 | Neoeriocitrin | 13241-32-2 |
|  |  |  | 柚皮苷 | Naringin | 10236-47-2 |
| 补骨脂 | *Psoralea corylifolia* L. | *Cullen corylifolium* (L.) Medik. | 补骨脂素 | Psoralen | 66-97-7 |
|  |  |  | 异补骨脂素 | Isopsoralen | 523-50-2 |
| 地黄 | *Rehmannia glutinosa* Libosch. | *Rehmannia glutinosa*(Gaertn.) DC | 梓醇 | Catalpol | 2415-24-9 |
|  |  |  | 地黄苷D | Rehmannioside D | 81720-08-3 |
| 羌活 | *Notopterygium incisum* Ting ex H.T.Chang | *Notopterygium incisum* K.C.Ting ex H.T.Chang | 羌活醇 | Notopterol | 88206-46-6 |
|  | *Notopterygium franchetii*H.de Boiss. | *Notopterygium franchetii* H.Boissieu | 异欧前胡素 | Isoimperatorin | 482-45-1 |
|  |  |  | 欧前胡素 | Imperatorin | 482-44-0 |
| 杜仲 | *Eucommia ulmoides* Oliv. | *Eucommia ulmoides* Oliv. | 松脂醇二葡萄糖苷 | Pinoresinol Diglucoside |  |
| 远志 | *Polygala tenuifolia* Willd. | *Polygala tenuifolia* Willd. |  | 3,6′-disinapoyl sucrose |  |
|  | *Polygala sibirica* L. | *Polygala sibirica* L. |  | Onjisaponin B |  |
